# PDGFRα reporter activity identifies periosteal progenitor cells critical for bone formation and fracture repair

**DOI:** 10.1038/s41413-021-00176-8

**Published:** 2022-01-25

**Authors:** Jiajia Xu, Yiyun Wang, Zhu Li, Ye Tian, Zhao Li, Amy Lu, Ching-Yun Hsu, Stefano Negri, Cammy Tang, Robert J. Tower, Carol Morris, Aaron W. James

**Affiliations:** 1 Departments of Pathology, Johns Hopkins University, Baltimore, MD 21205 USA; 2 Departments of Orthopedics, Johns Hopkins University, Baltimore, MD 21205 USA; 3 Department of Oral and Maxillofacial Surgery, School of Stomatology, China Medical University, Shenyang, Liaoning PR China

**Keywords:** Bone, Diseases

## Abstract

The outer coverings of the skeleton, which is also known as the periosteum, are arranged in concentric layers and act as a reservoir for tissue-specific bone progenitors. The cellular heterogeneity within this tissue depot is being increasingly recognized. Here, inducible PDGFRα reporter animals were found to mark a population of cells within the periosteum that act as a stem cell reservoir for periosteal appositional bone formation and fracture repair. During these processes, PDGFRα reporter^+^ progenitors give rise to Nestin^+^ periosteal cells before becoming osteoblasts and osteocytes. The diphtheria toxin-mediated ablation of PDGFRα reporter^+^ cells led to deficits in cortical bone formation during homeostasis and a diminutive hard callus during fracture repair. After ossicle transplantation, both mouse PDGFRα reporter^+^ periosteal cells and human Pdgfrα^+^ periosteal progenitors expand, ossify, and recruit marrow to a greater extent than their counterpart periosteal cells, whereas PDGFRα reporter^−^ periosteal cells exhibit a predisposition to chondrogenesis in vitro. Total RNA sequencing identified enrichment of the secreted factors *Fermt3* and *Ptpn6* within PDGFRα reporter^+^ periosteal cells, which partly underlie the osteoblastogenic features of this cell population.

## Introduction

The periosteum is a thin, dense layer of connective tissue that surrounds bones and is crucial to cortical bone homeostasis and repair.^1,2^ The periosteum contains poorly delineated skeletal precursor populations, which are anatomically separated into outer fibrous and inner cambium layers.^1,3,4^ Although the periosteum houses cells with two principal cellular fates (osteoblasts and chondroblasts), the discrimination of these progenitor cell subpopulations remains a challenge. At the early stage during embryonic endochondral ossification, the periosteum is formed from the perichondrium, which is rich in Nestin^+^ cells.^5^ Nestin^+^ and Lepr^+^ cells in the periosteum are subsets of periosteum-derived multipotent skeletal stem cells and possess multipotent and self-renewal abilities.^6^ Gli1-lineage cells in the periosteum rapidly expand in response to fracture and produce osteoblasts and/or chondrocytes.^7^ Mx1^+^αSMA^+^ cells also label a periosteal subpopulation with high colony formation potential.^8^ Cathepsin K^+^ periosteal cells highlight a periosteal population that is prone to bone but not cartilage formation.^9^ Markers such as PDPN, CD73, and CD164 have been described to identify self-renewing human skeletal stem cells within the periosteum.^10^ Platelet-derived growth factor receptor β (PDGFRβ) is a marker for skeletal stem and progenitor cells, including periosteal stem cells. PDGF-PDGFRβ signaling induces stem cell proliferation, trafficking, and angiotropism and mediates callus formation during bone repair.^11^

Platelet-derived growth factor receptor α (PDGFRα) is an emerging stem cell marker and can identify progenitor cell populations in multiple tissues, such as the periosteum, bone marrow, adipose tissue, and skeletal muscle.^12–15^ This marker has also been used to isolate Sca1^+^ stem cells from the heart and bone marrow.^12,16–18^ Platelet-derived growth factor (PDGF) is a potent mitogen for skeletal precursor cells and a critical mediator during early fracture healing.^19^ Periosteal progenitor cells express the PDGF receptor during fracture repair.^20^ Whether PDGFRα^+^ periosteal stem cells contribute to self-renewal and bone regeneration and are precursor cells of different subpopulations within this cellular niche remain important but largely unaddressed questions.

In this study, we utilized inducible PDGFRα reporter animals to identify a population of cells within the periosteum that act as a stem cell reservoir for periosteal appositional bone formation and fracture repair. Using cell ablation studies in combination with fluorescence-activated cell sorting (FACS) purification and transplantation, we found that PDGFRα reporter^+^ periosteal cells are a highly osteoblastogenic population responsible for the canonical functions of the periosteum during homeostasis and repair. Transcriptomics revealed the enrichment of two novel secreted factors within the PDGFRα reporter^+^ periosteum that underlie the osteoprogenitor features of this cell population.

## Results

### PDGFRα reporter activity marks skeletal progenitor cells within the periosteum

**Fig. 1:**
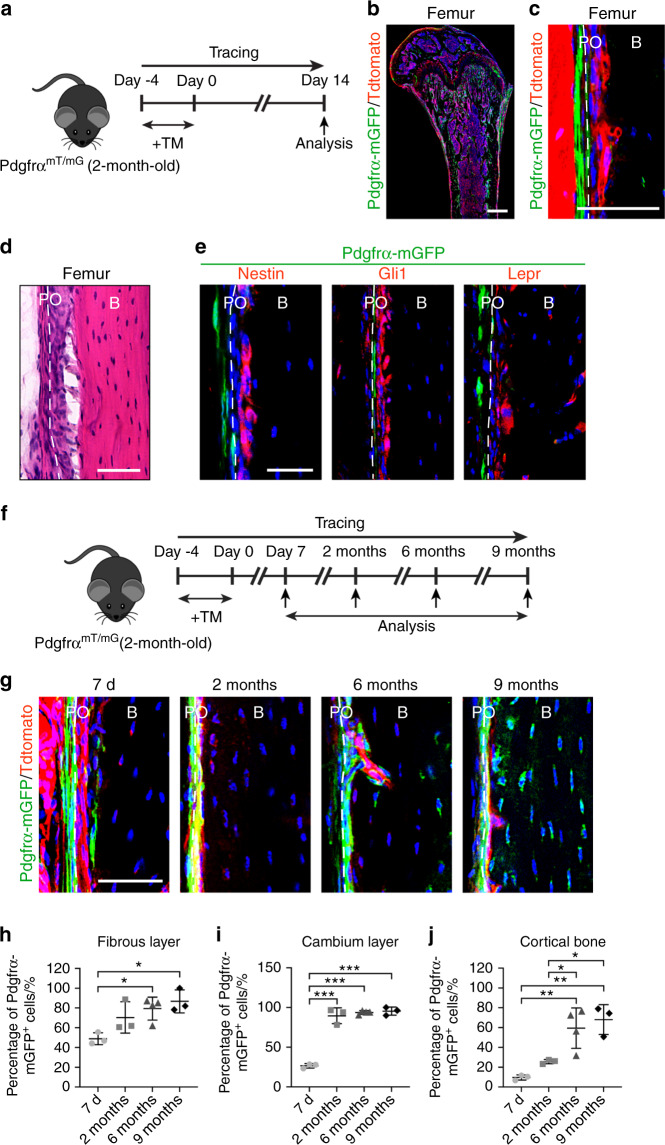
PDGFRα reporter activity marks skeletal progenitor cells within the periosteum. **a–e** PDGFRα reporter activity is mainly present in the fibrous layers of the periosteum. **a** Schematic of the experiment: PDGFRα^mT/mG^ animals (male, aged 2 mo) were administered tamoxifen (TM), and reporter activity was examined after 2 weeks. PDGFRα reporter^+^ (GFP^+^) cells were present in the femoral diaphyseal periosteum, as demonstrated by tile scanning (**b**) and at high magnification (**c**). Nuclei, DAPI (4′,6-diamidino-2-phenylindole, blue). **d** Representative histologic appearance of the femoral periosteum observed by H&E staining. **e** Immunohistochemical staining for Nestin, Gli1, and Lepr within the femoral diaphyseal periosteum using PDGFRα^mT/mG^ reporter sections and detection under a confocal microscope. GFP indicates reporter activity, and Tdtomato expression is not shown. **f–j** PDGFRα reporter^+^ periosteal cells give rise to the cambium layer of the periosteum and osteocytes over time. **f** Schematic of the experiment: PDGFRα^mT/mG^ animals (male, aged 2 mo) were administered TM, and reporter activity was examined after 7 d, 2 mo, 6 mo, and 9 mo. **g** Representative images of the PDGFRα^mT/mG^ diaphyseal periosteum at different time points after TM injection. Percentage of PDGFRα reporter^+^ cells in the fibrous periosteum (**h**), cambium periosteum (**i**), and osteocytes (**j**). B bone, PO periosteum. The dashed lines in **c**, **d**, **e**, and **g** indicate the limit between the inner and outer layers of the periosteum. Scale bars: 500 μm (**b**) and 50 μm (**c**, **d**, **e**, **g**). *n* = 3–4 animals per group. The dot plots represent an individual animal, and the whisker plots indicate the mean and one-SD values. *P < 0.05, **P < 0.01 and ***P < 0.001, as assessed by one-way ANOVA with Tukey’s multiple-comparisons test

The long bones of PDGFRα^mT/mG^ mice showed PDGFRα reporter activity within bone-lining cells, including the periosteum, endosteum and trabecular bone (Fig. 1a–c, Supplementary Fig. S1). The basal CreER^T2^ leakage levels in the absence of tamoxifen (TM) were very low in reporter mice (Supplementary Fig. S2). In the periosteum, PDGFRα reporter activity was primarily found within the outer fibrous layer (Fig. 1c, d), whereas immunohistochemical detection of other ‘skeletal stem cell’ antigens, including Nestin, Gli1, and Lepr, was primarily found in the inner cambium layer (Fig. 1e; the coexpression frequency is indicated in Supplementary Fig. S3). Although PDGFRα reporter activity has been found in perivascular areas in other tissues,^14^ this finding was not clearly observed in the periosteum (Supplementary Fig. S4). The numbers of PDGFRα reporter^+^ periosteal cells gradually decreased with increases in the mouse age (Supplementary Fig. S5), although at least some periosteal reporter activity was detected at all ages.

The fibrous periosteum has long been hypothesized to serve as a stem cell niche for replenishing the cambium and cortical bone.^21^ To assess the cell fate of PDGFRα reporter^+^ cells within the periosteum, the femurs of PDGFRα^mT/mG^ mice were analyzed during long-term chasing after TM administration (up to 9 mo later, Fig. 1f, g). A gradual increase in reporter activity was observed over time in the fibrous periosteum, and 41.6% to 86.7% of cells within this tissue layer exhibited this activity (Fig. 1h). Similarly, a significant increase over time was observed in the cambium layer, and up to 95.5% of the cells exhibited this activity at the study endpoint (Fig. 1i). The proportion of osteocytes lying adjacent to the periosteum that exhibited mGFP expression also showed a prominent increase, from 9.7% of cells at 7 d to 68.1% of cells at 9 mo (Fig. 1j). Osteocytes lying deeper within cortical bone and adjacent to the endosteum demonstrated a similar trend over time (*not shown*).

**Fig. 2:**
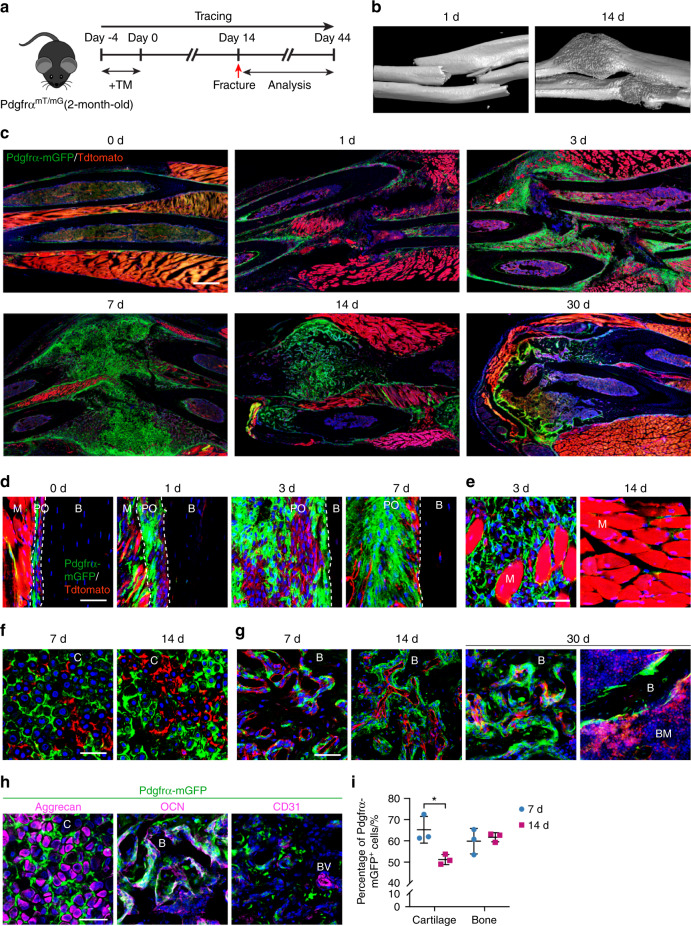
PDGFRα reporter^+^ periosteal cells give rise to osteochondral cells within a fractured callus. **a** PDGFRα^mT/mG^ reporter animals (male, aged 2 mo) were administered TM, forelimb fracture was performed after 14 d, and an analysis was performed 1–30 d later. **b** Micro-CT 3D reconstructions at representative time points after fracture. **c** Representative tile scans of the whole forelimb at serial time points after fracture ranging from 0 to 30 d. The uninjured forelimb is shown for comparison. Nuclei, DAPI (4′,6-diamidino-2-phenylindole, blue). **d** Representative high-magnification images of PDGFRα reporter activity in the periosteum of unfractured or fractured bone (d 1–7). **e** High-magnification images of GFP^+^ cells in skeletal muscle adjacent to the fracture site (3 and 14 d). The dashed lines indicate the limit between the periosteum and muscle or bone. **f** Contribution of GFP^+^ cells in the cartilage of the fractured callus (7 and 14 d). **g** Contribution of GFP^+^ cells to new osteoblasts in the fractured callus (7–30 d). **h** Representative immunohistochemical staining of a PDGFRα^mT/mG^ fractured callus was performed 7 or 14 d after injury, including staining for aggrecan, osteocalcin (OCN), and CD31. **i** Percentages of mGFP^+^ chondrocytes/total chondrocytes and mGFP^+^ osteoblasts/total osteoblasts assessed at 7 and 14 d after injury. B bone, BM bone marrow, BV blood vessel, C cartilage, M skeletal muscle, PO periosteum. Scale bars: 500 μm (**c**) and 50 μm (**d**–**h**). *n* = 3 animals per group. The dot plots represent an individual animal, and the whisker plots indicate the mean and one-SD values. *P < 0.05, as assessed by two-tailed Student’s *t* tests

A similarly prominent generation of skeletal cells from PDGFRα reporter^+^ cells was observed after fracture (Fig. 2). Here, a closed, nonstabilized fracture in the mid-diaphysis of the forelimbs was examined over a 30-d period (Fig. 2a–i). A significant increase in periosteal PDGFRα reporter activity was noted as early as 1 d after fracture (Fig. 2c, d) and gave rise to most of the fracture callus at later timepoints (Fig. 2c). Robust expansion of PDGFRα reporter^+^ periosteal cells was observed over time (Fig. 2d), whereas a transient expansion of PDGFRα reporter^+^ cells was detected within the interstitium of skeletal muscle next to the injury site (Fig. 2e). At later timepoints, a large fraction of chondrocytes within the fracture callus were mGFP^+^ (Fig. 2f), and the majority of bone-lining cells were also mGFP^+^ (Fig. 2g, see also Fig. 2i for quantification). The descendants of PDGFRα reporter^+^ cells were confirmed to give rise to both aggrecan^+^ chondrocytes and osteocalcin (OCN)^+^ osteoblasts but not CD31^+^ endothelial cells within the fracture callus (Fig. 2h). These histological observations were confirmed by flow cytometry for aggrecan, osteopontin and CD31 (Supplementary Fig. S6). In all fracture studies, minimal recombination in the absence of TM administration was observed (*not shown*). These data indicate that PDGFRα reporter activity marks a major skeletal progenitor pool within the periosteum, and this pool contributes to both cortical bone renewal and fracture healing postnatally in mice.

### The depletion of PDGFRα reporter^+^ progenitor cells impairs cortical homeostasis and fracture repair

**Fig. 3:**
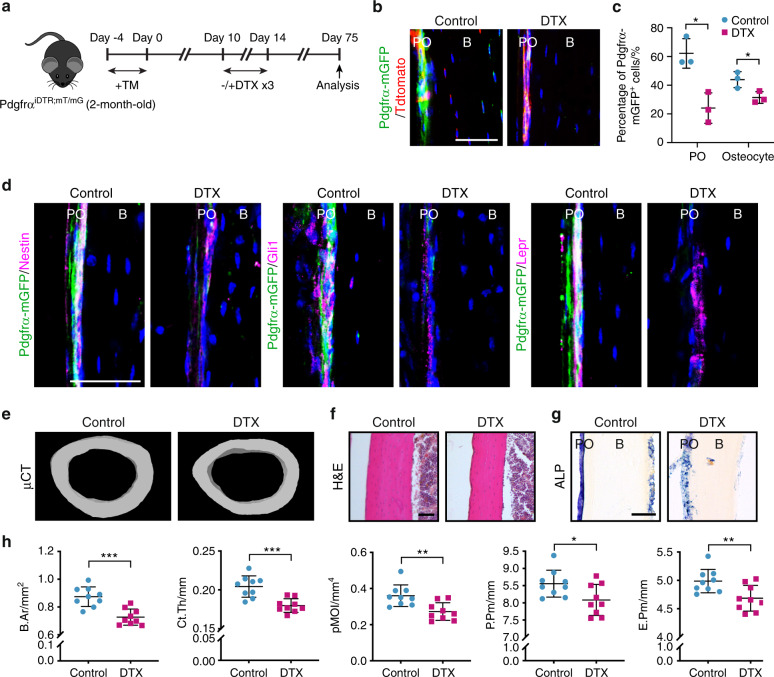
Depletion of periosteal PDGFRα reporter^+^ progenitor cells impairs cortical homeostasis. **a** PDGFRα^iDTR;mT/mG^ animals (male, aged 2 mo) were sequentially administered tamoxifen (TM) and diphtheria toxin (DTX) and analyzed after 2 mo. **b** Representative images of PDGFRα reporter activity (GFP) among DTX- or control-treated PDGFRα^iDTR;mT/mG^ animals. Nuclei, DAPI (4′,6-diamidino-2-phenylindole, blue). **c** Percentage of mGFP^+^ periosteal cells/total periosteal cells and mGFP^+^ osteocytes/total osteocytes. **d** Analysis of Nestin, Gli1, and LepR expression in DTX- or control-treated PDGFRα^iDTR;mT/mG^ animals by immunohistochemistry. **e** Micro-CT reconstructions of cortical bone in the midshaft femur. **f**, **g** Representative histologic appearance of the midshaft femur cortical bone observed by H&E (**f**) and alkaline phosphatase (ALP) staining (**g**). **h** Quantitative micro-CT analysis of the cortical bone in the control- and DTX-treated PDGFRα^iDTR;mT/mG^ animals at the femoral midshaft. The analysis included the bone area (B.Ar), cortical thickness (Ct.Th), polar moment of inertia (pMOI), periosteal perimeter (P.Pm), and endosteal perimeter (E.Pm). Scale bars: 50 μm. B bone, PO periosteum. The dot plots represent an individual animal, and the whisker plots indicate the mean and one-SD values. *n* = 3 animals per group (**b**–**d**) and 9 animals per group (**e**–**h**). *P < 0.05, **P < 0.01, and ***P < 0.001, as assessed by two-tailed Student’s *t* tests

To investigate the role of PDGFRα reporter^+^ progenitor cells in periosteal cortical homeostasis and bone turnover, PDGFRα reporter^+^ cells were genetically ablated by crossing PDGFRα^mT/mG^ mice with iDTR mice to generate PDGFRα^iDTR;mT/mG^ animals (Fig. 3a). After diphtheria toxin (DTX) injection, ablation of the majority of femoral PDGFRα reporter^+^ periosteal cells was observed (Fig. 3b, c), and a parallel reduction in mGFP^+^ osteocytes was also detected over time (Fig. 3c). Similarly, the ablation of PDGFRα reporter^+^ cells at 2 mo after DTX treatment led to reductions in the cellular contents within the cambium layer periosteum, and significant reductions were detected in the numbers of PDGFRα reporter^+^ progenitor cells that gave rise to Nestin-, Gli1-, or Lepr-immunoreactive cells (Fig. 3d, quantification is shown in Supplementary Fig. S7). A similar efficiency of cell ablation was noted within the endosteum 2 mo after DTX injection (Supplementary Fig. S8). The depletion of PDGFRα reporter^+^ progenitor cells resulted in a markedly thinner femoral cortex (Fig. 3e, f) and reduced periosteal alkaline phosphatase (ALP) activity (Fig. 3g). A quantitative micro-CT analysis confirmed that the DTX-treated group exhibited relatively lower values of several cortical bone-related parameters, including the bone area (B.Ar), cortical thickness (Ct.Th), bone perimeter (B.Pm), polar moment of inertia (pMOI), and periosteal and endosteal perimeters (P.Pm and E.Pm, respectively), than the control group (Fig. 3h). Similar results were found in the forelimb, and the ulna demonstrated reduced values of the cortical bone mass, thickness, and ALP activity (Supplementary Fig. S9). Taken together, these results indicate that PDGFRα reporter^+^ cells are needed for cortical bone homeostasis.

**Fig. 4:**
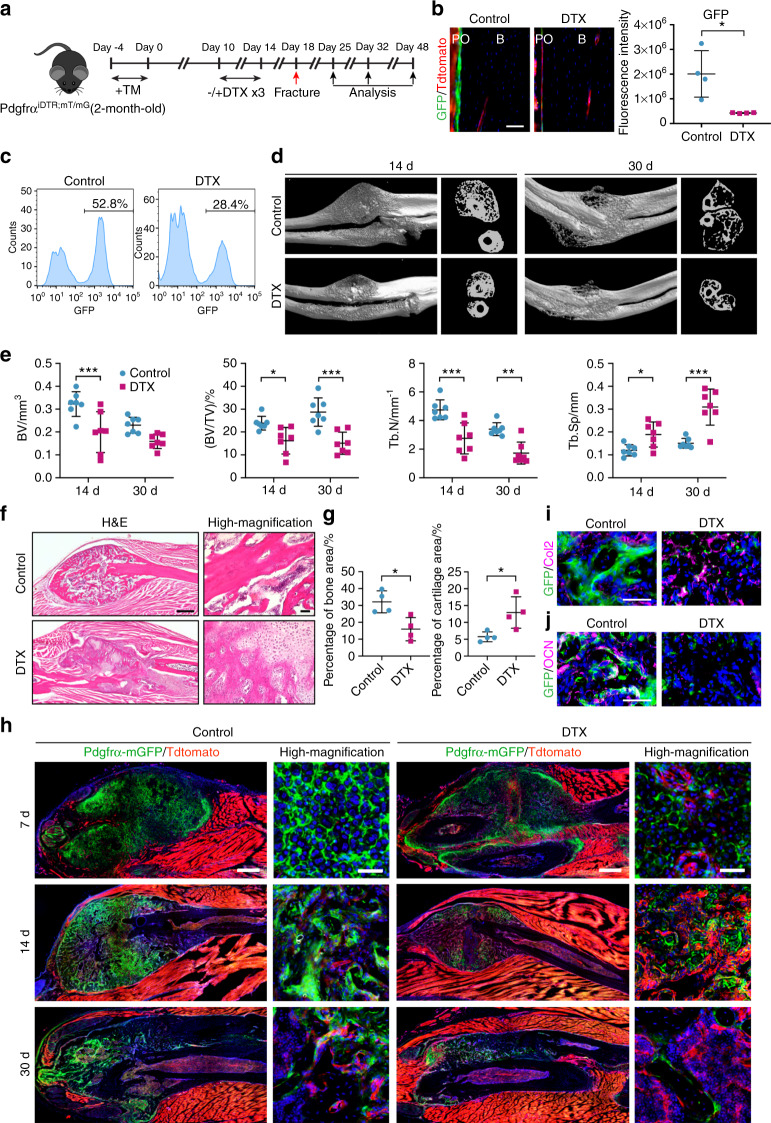
The depletion of periosteal PDGFRα reporter^+^ progenitor cells impairs fracture repair. **a** PDGFRα^iDTR;mT/mG^ animals were injected with TM and DTX, and forelimb fractures were then performed. The analysis was performed 7, 14, and 30 d after fracture. **b** Efficiency of cell depletion in the periosteum, as shown by representative images and histologic quantification of PDGFRα reporter activity (GFP) 14 d after DTX injection. Nuclei, DAPI (4′,6-diamidino-2-phenylindole, blue). **c** Efficiency of PDGFRα reporter^+^ cell depletion determined by flow cytometry 14 d after DTX injection. **d** Representative micro-CT 3D reconstructions and axial cross-sectional images obtained 14 and 30 d after fracture. **e** Quantitative analysis of micro-CT images obtained at 14 and 30 d after fracture. The analysis included the bone volume (BV), fractional bone volume (BV/TV), trabecular number (Tb.N), and trabecular spacing (Tb.Sp). **f** Representative H&E-stained images of the fractured callus in PDGFRα^iDTR;mT/mG^ animals 14 d after fracture. **g** Percentages of bone and cartilage within the total callus area 14 d after fracture. **h** Representative tile scans and high-magnification images of the fracture site and associated callus in PDGFRα^iDTR;mT/mG^ reporter animals at 7, 14 and 30 d after fracture. Immunohistochemical staining of PDGFRα^iDTR;mT/mG^ fractured calluses with or without DTX 14 d after injury, including (**i**) collagen type 2 (Col2) and (**j**) osteocalcin (OCN). Positive immunohistochemistry is shown in purple, and PDGFRα reporter activity (GFP) appears green. Scale bars: 500 μm (**f**, **h** (tile scan)) and 50 μm (**b**, **f**, **h**–**j**). B bone, PO periosteum. The dot plots represent an individual animal, and the whisker plots indicate the mean and one-SD values. *n* = 4 animals per group (**b**, **c**, **f**–**j**) and *N* = 7 animals per group (**d**, **e**). *P < 0.05, **P < 0.01, and ***P < 0.001, as assessed by two-tailed Student’s *t* tests

To further confirm the importance of PDGFRα reporter^+^ progenitor cells in the established functions of the periosteum, DTX-mediated ablation was then performed prior to a closed, nonstabilized fracture in PDGFRα^iDTR;mT/mG^ mice (Fig. 4a). Cell depletion efficiency was again confirmed via immunofluorescent detection of reporter activity (Fig. 4b) and flow cytometry (Fig. 4c). The depletion of PDGFRα reporter^+^ cells diminished the callus size and ossification, as assessed by micro-CT at 14 and 30 d after fracture (Fig. 4d). The quantification of micro-CT images confirmed this impression, as demonstrated by reductions in the bone volume (BV), bone volume fraction (BV/TV), and trabecular number (Tb.N) after DTX treatment and a converse increase in the trabecular spacing (Tb.Sp) (Fig. 4e). Hematoxylin and eosin (H&E) staining revealed distinctive features of impaired bone formation in the DTX-treated group, and these features included a decreased bone area and an increase in remnant cartilage at 14 d after fracture (Fig. 4f, g). H&E images indicated more frequent mature trabeculae of lamellar bone among the control animals, whereas more apparent areas of immature woven bone were detected within the fracture sites of DTX-treated animals (Supplementary Fig. S10). Tissue sections of fractured calluses (7–30 d) confirmed a significant depletion of mGFP^+^ cells and a reduced callus size among the DTX-treated animals (Fig. 4h). Immunofluorescence staining confirmed these findings, which demonstrated by more obvious type 2 collagen (Col2)^+^ chondrocytes among the DTX-treated animals at 14 d after fracture (Fig. 4i). Conversely, more obvious OCN^+^ osteoblasts were found among the control group (Fig. 4j). In total, these data indicate that PDGFRα reporter^+^ progenitor cells are essential for cortical bone homeostasis and fracture repair.

### Mouse PDGFRα reporter^+^ and human PDGFRα^+^ periosteal progenitors have high osteogenic potential

**Fig. 5:**
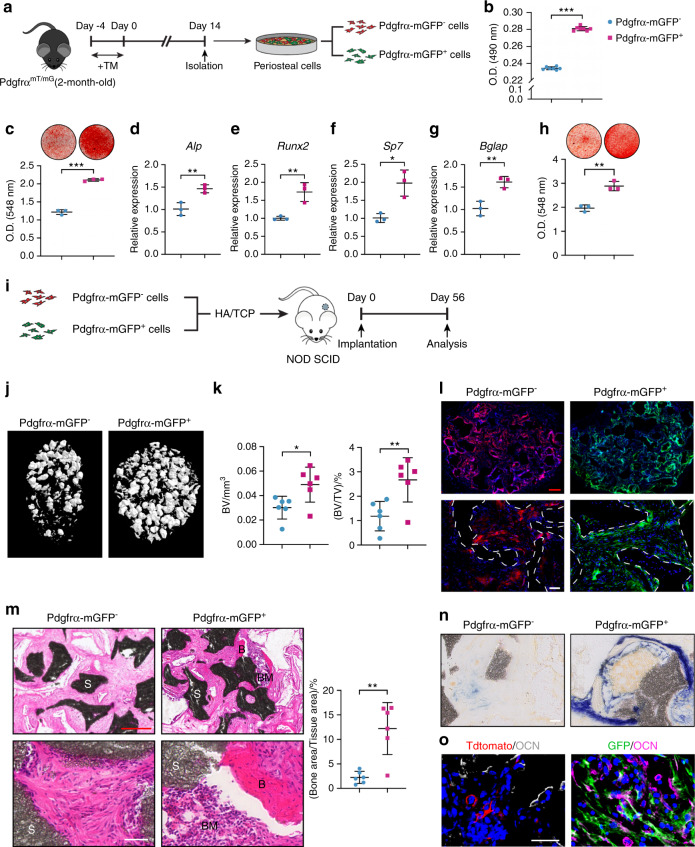
Mouse PDGFRα reporter^+^ periosteal progenitors have high osteogenic potential. **a** Schematic of experiments in which PDGFRα reporter^−^CD31^−^CD45^−^Ter119^−^ and PDGFRα reporter^+^CD31^−^CD45^−^Ter119^−^ periosteal cells were derived by FACS from PDGFRα^mT/mG^ hindlimbs (male, aged 2 mo). **b** Cell proliferation among PDGFRα reporter^−^ and PDGFRα reporter^+^ periosteal cells determined by MTS assays at 72 h. **c** Osteogenic differentiation of PDGFRα reporter^−/+^ periosteal cells at 16 d determined by Alizarin Red (AR) staining and quantification. Expression of the osteogenic genes, including (**d**) *alkaline phosphatase* (*Alp*), (**e**) *runt-related transcription factor 2* (*Runx2*), (**f**) *Osterix* (*Sp7*), and (**g**) *osteocalcin* (*Bglap*), in PDGFRα reporter^−/+^ periosteal cells after 7 d of differentiation. **h** Osteogenic differentiation of freshly isolated PDGFRα reporter^−/+^ periosteal cells at 14 d determined by Alizarin Red (AR) staining and quantification. **i** Schematic of ossicle assay. PDGFRα reporter^−^ or reporter^+^ cells (3 × 10^6^) were subcutaneously implanted into NOD SCID mice using an HA/β-TCP carrier and harvested after 8 weeks. **j** Representative micro-CT reconstructions of the implants. **k** Quantitative analysis of micro-CT images of PDGFRα reporter^−/+^ periosteal cells. The analysis included the bone volume (BV) and fractional bone volume (BV/TV). **l** Persistence of PDGFRα reporter^+^ and PDGFRα reporter^−^ periosteal cells. PDGFRα reporter^−^ cells appeared red, and PDGFRα reporter^+^ cells appeared green. Nuclei, DAPI (4′,6-diamidino-2-phenylindole, blue). The white dashed lines demarcate the edges of the scaffold. **m** Representative histologic appearance determined by H&E, including low-magnification (above left) and high-magnification (below left) images, and quantification of the bone area/tissue area (right). **n** Representative ALP staining. **o** OCN immunohistochemistry. Positive immunohistochemistry is shown by purple or white. PDGFRα reporter^−^ cells appeared red (Tdtomato^+^), and PDGFRα reporter^+^ cells appeared green (GFP^+^). Red scale bars: 500 μm; White scale bars: 50 μm. B bone, BM bone marrow, S scaffold. The dot plots represent an individual sample or animal, and the whisker plots indicate the mean and one-SD values. In vitro experiments were performed in biological and experimental triplicates (**a**–**h**). For subcutaneous implantation, *n* = 6 animals per group (**i**–**o**). *P < 0.05, **P < 0.01 and ***P < 0.001, as assessed by two-tailed Student’s *t* tests

FACS analysis of the periosteum of 2-mo-old PDGFRα^mT/mG^ mice showed that an average of 50.8% of CD31^−^CD45^−^Ter119^−^ periosteal cells was positive for the PDGFRα reporter (Supplementary Table S1, Supplementary Fig. S11). After culture expansion (Fig. 5a), PDGFRα reporter^+^ periosteal cells demonstrated a higher proliferative rate (as assessed by MTS assays; Fig. 5b) and enhanced osteogenic potential in comparison to their PDGFRα reporter^−^ periosteal cell counterparts (Fig. 5c–g). Under osteogenic differentiation conditions, although both cell populations were able to form bone nodules, PDGFRα reporter^+^ periosteal cells formed significantly greater bone nodules, as visualized by Alizarin red staining (Fig. 5c). Additionally, an enrichment of osteogenic gene transcripts was also observed among PDGFRα reporter^+^ periosteal cells, and these transcripts included *Alp* (Fig. 5d), *runt-related transcription factor 2* (*Runx2*) (Fig. 5e), *Osterix* (*Sp7*) (Fig. 5f) and *Bglap* (Fig. 5g). Similar osteogenic differences were also observed among freshly isolated and FACS-identified PDGFRα reporter^+/−^ cells without preculture expansion (Fig. 5h). In contrast, PDGFRα reporter^−^ periosteal cells displayed an increase in the expression of cartilage-associated genes, such as *Sox9*, *Acan*, *Comp*, and *Col2a1* (Supplementary Fig. S12a–d), and more apparent chondrogenic differentiation in three-dimensional micromass culture (Supplementary Fig. S12e, f). Based on in vitro differentiation studies, PDGFRα reporter^+^ cells displayed an increased predisposition to mineralization, whereas PDGFRα reporter^−^ cells exhibited a predisposition to chondrogenesis.

Ossicle formation assays were then performed using purified PDGFRα reporter^+/−^ periosteal cells by subcutaneous implantation in NOD SCID mice (6 transplants per group, 3 million cells mixed with hydroxyapatite/β-tricalcium phosphate scaffold) (Fig. 5i). Micro-CT reconstructions showed that PDGFRα reporter^+^ periosteal cells led to significantly greater bone formation in vivo (Fig. 5j). The micro-CT quantification of BV and BV/TV confirmed significant increases among PDGFRα reporter^+^ cell implants (Fig. 5k). Both periosteal cell fractions demonstrated clear engraftment and persistence within the implant site (Fig. 5l). H&E staining confirmed more apparent new bone formation among PDGFRα reporter^+^ periosteal implants, including a 443% increase in the bone area/tissue area ratio (Fig. 5m). Mineralized bone was apparent in 5 of 6 PDGFRα reporter^+^ implants in comparison to 1 of 6 PDGFRα reporter^−^ implants. Similarly, bone marrow recruitment was identified in 1 of 6 PDGFRα reporter^+^ implants but not in any PDGFRα reporter^−^ implants. Staining for ALP enzymatic activity also demonstrated clear differences, with abundant ALP activity in the PDGFRα reporter^+^ periosteal implants (Fig. 5n). The immunofluorescent detection of OCN demonstrated robust coexpression with GFP among reporter^+^ implants, and less apparent OCN immunoreactivity was detected among Tdtomato^+^ cells in the PDGFRα reporter^−^ implant sites (Fig. 5o).

**Fig. 6:**
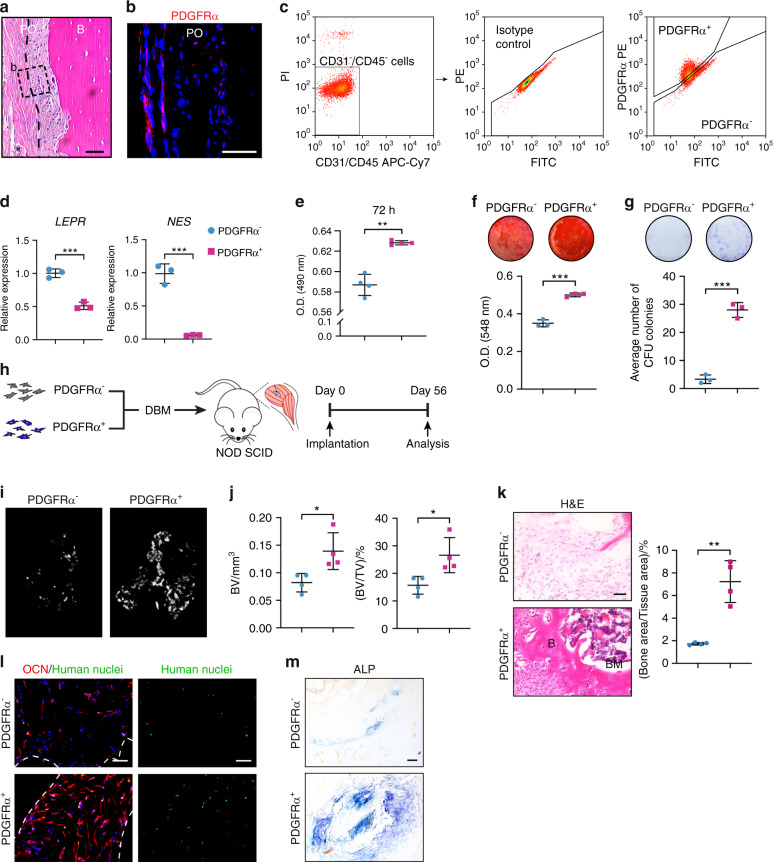
Human PDGFRα^+^ periosteal progenitors have high osteogenic potential. **a** Representative histologic appearance of the human periosteum observed by H&E staining. The dashed line indicates the limit between the inner and outer layers of the periosteum. The dashed box indicates the location of **b**. **b** PDGFRα immunohistochemistry (shown in red) in human diaphyseal periosteum (*n* = 3). Nuclei, DAPI (blue). **c** PDGFRα^−^ and PDGFRα^+^ cell isolation from the human diaphyseal periosteum. PDGFRα^−^ and PDGFRα^+^ cells were isolated from the CD31^−^CD45^−^ nonendothelial/nonhematopoietic cell population. Isotype control staining is shown. **d** Progenitor cell markers among human PDGFRα^−^ and PDGFRα^+^ stromal cells from the same patient sample, including *leptin receptor* (*LEPR*) and *Nestin* (*NES*), were detected by qRT–PCR. **e** Cellular proliferation among human PDGFRα^−^ and PDGFRα^+^ periosteal cells at 72 h as assessed by the MTS assay. **f** Osteogenic differentiation of PDGFRα^−^ and PDGFRα^+^ periosteal cells as assessed by Alizarin Red (AR) staining. **g** Fibroblast colony-forming units (CFU-Fs) among human PDGFRα^−^ and PDGFRα^+^ periosteal cells. **h** Schematic of the ossicle assay. PDGFRα^−^ or PDGFRα^+^ cells (3 × 10^6^) were intramuscularly implanted into NOD SCID mice using a DBX carrier and harvested 8-week later. **i** Micro-CT reconstructions of the implants. **j** Quantitative analysis of the micro-CT images of human PDGFRα^−^ and PDGFRα^+^ stromal cells. The analysis included the bone volume (BV) and fractional bone volume (BV/TV). **k** Representative histologic appearance determined by H&E staining (left) and quantification of the bone area/tissue area (right). **l** Immunofluorescence staining of osteocalcin (OCN, red) and human-specific nuclei (green) within implants laden with PDGFRα^−^ or PDGFRα^+^ cells. The white dashed lines demarcated the edges of the scaffold. **m** Representative ALP staining. Scale bars: 50 μm. B bone, BM bone marrow, PO periosteum. The dot plots represent an individual sample or animal, and the whisker plots indicate the mean and one-SD values. The experiments were performed in triplicate. *n* = 4 animals per group. *P < 0.05, **P < 0.01 and ***P < 0.001, as assessed by two-tailed Student’s *t* tests

Subpopulations of human microdissected periosteum from the femur and tibia were then examined based on PDGFRα expression (Fig. 6). As in the mouse periosteum, PDGFRα-immunoreactive cells were most frequently detected in the outer fibrous periosteum of human long bone (Fig. 6a, b). Microdissected and dissociated periosteum underwent lineage depletion and FACS purification to obtain PDGFRα^+^ and PDGFRα^−^ cell populations (Fig. 6c). Consistent with the findings from the mouse periosteum, both the *LEPR* and *NES* markers were enriched in the PDGFRα^−^ periosteal cell fraction (Fig. 6d). Various cellular parameters were then assessed among culture-expanded PDGFRα^−^ and PDGFRα^+^ periosteal cell preparations (Fig. 6e–g). Similar to the findings obtained for the mouse periosteum, culture-expanded PDGFRα^+^ periosteal cell populations showed increased proliferation (Fig. 6e), enhanced osteogenic differentiation (Fig. 6f), and a higher frequency of fibroblast colony-forming units (CFU-Fs) (Fig. 6g) in comparison to the PDGFRα^−^ periosteal cells. These observed differences in the osteogenic potential of human PDGFRα^+^ versus PDGFRα^−^ periosteal cells were then assayed in vivo. For this purpose, culture-expanded human PDGFRα^−^ and PDGFRα^+^ periosteal cells from the same sample were implanted in equal numbers (4 transplants per group, 3 million cells mixed with demineralized bone matrix scaffold) into the thigh muscle pouch in NOD SCID mice (Fig. 6h). After 8 weeks, significant de novo bone formation was observed in PDGFRα^+^ periosteal implants, as observed by micro-CT reconstructions of the implant site (Fig. 6i). Quantitative analysis confirmed increases in BV and BV/TV (Fig. 6j). H&E staining demonstrated more apparent new bone formation in PDGFRα^+^ implants, including a 317% increase in the bone area/tissue area ratio (Fig. 6k). Mineralized bone was observed in 4 of 4 PDGFRα^+^ implants but only in 1 of 4 PDGFRα^−^ implants. Bone marrow was found in 3 of 4 PDGFRα^+^ implants but only observed in 1 of 4 PDGFRα^−^ implants. The detection of human nuclear antigen among implant sites confirmed the persistence of human cells across both groups (Fig. 6l). OCN immunofluorescence and ALP staining confirmed the enrichment of these osteoblast antigens among PDGFRα^+^ periosteal implants (Fig. 6l, m). Thus, the PDGFRα-expressing cell fraction in the human periosteum represents a precursor population with a higher basal ability to form bone than the non-Pdgfrα-expressing cell fraction.

### PDGFRα reporter^+^ periosteal progenitors give rise to Nestin^+^ periosteal cells during osteogenesis

Within the mouse long bone, a clear anatomical separation was observed between the PDGFRα reporter^+^ fibrous periosteum and a Nestin-expressing inner cambium layer. To further investigate the relationship among cell types, FACS-defined mouse PDGFRα reporter^+^ periosteal cells were exposed to osteogenic differentiation conditions. Transcripts of both *Lepr* and *Nes* were significantly induced upon exposure to osteogenic differentiation conditions (Supplementary Fig. S13a). The hypothesis that PDGFRα reporter^+^ periosteal cells give rise to Nestin^+^ cells during bone formation in vivo was evaluated by the Nestin immunohistochemistry analysis of previously obtained PDGFRα^mT/mG^ reporter samples (Supplementary Fig. S13b–h). After long-term chase periods in the uninjured femoral periosteum of PDGFRα^mT/mG^ mice, unipositive PDGFRα reporter^+^ periosteal cells expanded from the outer layer to the inner periosteal layer to become mGFP^+^Nestin^+^ double-positive cells (Supplementary Fig. S13b, c). Similar findings were observed after long bone fracture (Supplementary Fig. S13d–f). At early timepoints after fracture, unipositive mGFP^+^ cells were most common (3 d shown). As bone matrix appeared, increased colocalization of mGFP^+^ and Nestin^+^ cells was identified on bone-lining surfaces (7 and 14 d shown). Moreover, the generation of Nestin^+^ cells was further confirmed among subcutaneous implants of either PDGFRα reporter^+^ or PDGFRα reporter^−^ mouse periosteal cells (Supplementary Fig. S13g–i). Here, the transplantation of PDGFRα reporter^+^ periosteal cells gave rise to high numbers of mGFP^+^Nestin^+^ cells, whereas the PDGFRα reporter^−^ periosteal cell implants induced the generation of lower numbers of these cells (Supplementary Fig. S13h, i). Thus, PDGFRα reporter^+^ periosteal cells acquire Nestin expression during the bone formation, and the findings suggest a cellular hierarchy among periosteal cell derivatives.

### Fermt3 and Ptpn6 underlie the osteoblastogenic features of PDGFRα reporter^+^ periosteal progenitors

**Fig. 7:**
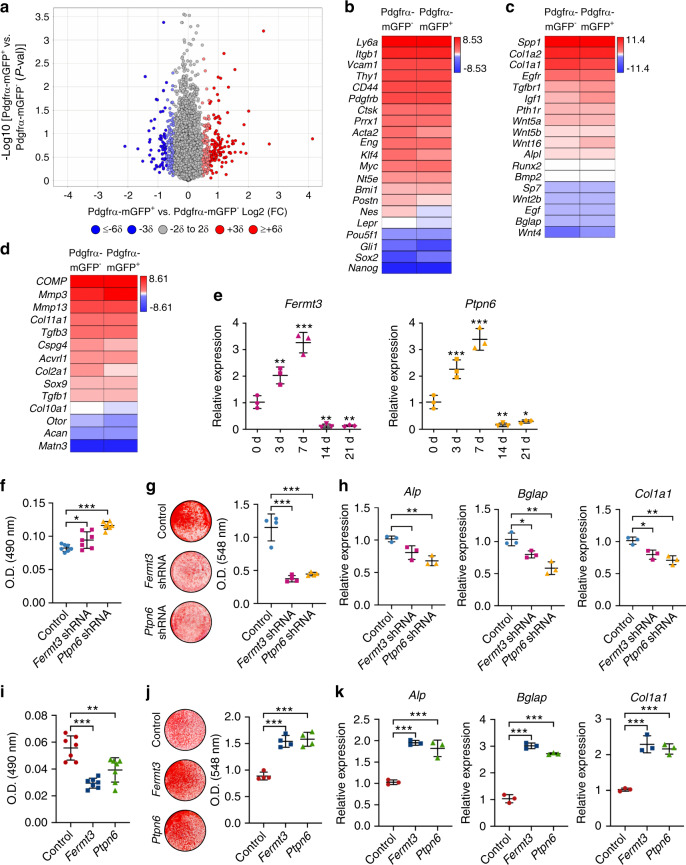
*Fermt3* and *Ptpn6* gene enrichment underlies the osteoblastic phenotype of PDGFRα reporter^+^ periosteal cells. **a–d** Total RNA sequencing comparison between PDGFRα reporter^−^CD31^−^CD45^−^Ter119^−^ and PDGFRα reporter^+^CD31^−^CD45^−^Ter119^−^ cells from the mouse periosteum of PDGFRα^mT/mG^ animals. **a** Volcano plot of all transcripts. The *x*-axis represents the log2(fold change) for each gene. The *y*-axis represents the -log10(*P* value). The red dots indicate a > 2 SD increase among PDGFRα reporter^+^ periosteal cells, and the blue dots indicate a > 2 SD increase among PDGFRα reporter^−^ periosteal cells. **b** Heatmap demonstrating the mRNA expression levels of stemness- and stem cell-related markers among PDGFRα reporter^+^ and PDGFRα reporter^−^ periosteal cells. Heatmap of the expression of (**c**) osteogenic gene markers and (**d**) chondrogenic gene markers among PDGFRα reporter^+^ and PDGFRα reporter^−^ periosteal cells. **e** Relative expression of *Fermt3* and *Ptpn6* at the indicated time points during osteogenesis detected by qRT–PCR. The differences are shown in comparison to the initial expression (at d 0). **f** Cell proliferation of PDGFRα reporter^+^ periosteal cells with or without *Fermt3* or *Ptpn6* shRNA determined by MTS assays (48 h). Osteogenic differentiation of PDGFRα reporter^+^ periosteal cells with or without *Fermt3* or *Ptpn6* shRNA assessed (**g**) by Alizarin Red (AR) staining (11 d) and (**h**) based on the expression of osteogenic genes, including *alkaline phosphatase* (*Alp*), *osteocalcin* (*Bglap*), and *collagen type 1 a1* (*Col1a1*) (7 d), measured by qRT–PCR. **i** Cell proliferation of PDGFRα reporter^−^ periosteal cells with or without *Fermt3* or *Ptpn6* overexpression measured by MTS assays (48 h). Osteogenic differentiation of PDGFRα reporter^−^ periosteal cells with or without *Fermt3* or *Ptpn6* overexpression assessed (**j**) by AR staining (8 d) and (**k**) based on osteogenic gene expression determined by qRT–PCR (7 d). The dot plots represent an individual sample, and the whisker plots indicate the mean and one-SD values. *P < 0.05, **P < 0.01 and ***P < 0.001, as assessed by one-way ANOVA with Tukey’s multiple-comparisons test

To further define the differences among periosteal cell subpopulations, periosteal cells were isolated and briefly cultured. The overall osteogenic differences were observed with or without bFGF supplementation during culture expansion. The transcriptome of mouse FACS-identified PDGFRα reporter^−^ and PDGFRα reporter^+^ periosteal cells was evaluated by bulk RNA sequencing after the removal of CD31-, CD45-, and Ter119-expressing cells (Fig. 7). A total of 10 327 genes were expressed in all of the samples and had functional annotations. A total of 247 transcripts (2.39% of the total) showed a > 2 standard deviation (SD) increase among PDGFRα reporter^+^ cells [red dots; 29 transcripts showed a significant increase (*P* < 0.05)], and 193 transcripts (1.87% of the total) showed a > 2 SD decrease among PDGFRα reporter^+^ cells [blue dots; 22 transcripts showed a significant decrease (*P* < 0.05)] (Fig. 7a). Endothelial and inflammatory marker genes were rarely or not expressed among either cell population, which further confirmed the success of our FACS purification (Supplementary Fig. S14a, b). Both PDGFRα reporter^−^ and PDGFRα reporter^+^ periosteal cells shared the expression of stemness-associated genes and canonical bone marrow stromal cell markers, including *Myc* (MYC proto-oncogene, BHLH transcription factor), *Bmi1* (BMI1 proto-oncogene, polycomb ring finger), *Pou5f1* (POU class 5 homeobox 1), *CD44*, *THY1* (CD90), and *Ly6a* (stem cell antigen-1), although some subtle differences were noted. For example, *Klf4* (Krüppel-like factor 4), *Nes*, and *Lepr* were enriched in PDGFRα reporter^−^ cells, whereas PDGFRα reporter^+^ periosteal cells expressed higher levels of *Sox2* (sex-determining region Y-box 2) (Fig. 7b). *Pdgfrβ* (platelet-derived growth factor receptor β), *Ctsk* (cathepsin K), and *Acta2* (actin alpha 2, smooth muscle) were highly expressed in both periosteal cell populations, whereas *Gli1* was expressed at low levels in both cell preparations. QIAGEN Ingenuity Pathway Analysis (IPA) showed that the activated pathways in PDGFRα reporter^+^ stromal cells are associated with the positive regulation of osteogenesis, including Toll-like receptor signaling, estrogen receptor signaling, and endothelin-1 signaling (Z scores of 2, 1.941, and 1.342, respectively; Supplementary Fig. S14c).^22–24^ Conversely, the upregulated signaling pathways in PDGFRα reporter^−^ periosteal cells were associated with collagen expression and the positive regulation of chondrogenic differentiation, including the GP6 signaling pathway, p53 signaling and PI3K/AKT signaling (Z scores of −1.732, −1.342, and −1, respectively; Supplementary Fig. S14c).^25–27^ Genes associated with osteogenic and chondrogenic cell fate decisions were then assessed (Fig. 7c, d). Selected genes associated with osteogenic differentiation, such as *Tgfbr1* (transforming growth factor-β receptor type 1), *Igf1* (insulin-like growth factor 1), *Wnt4* (Wnt family member 4), and *Wnt16*, were more highly expressed among PDGFRα reporter^+^ periosteal cells (Fig. 7c).^28–31^ Conversely, gene markers associated with chondrogenic differentiation, including *Col2a1* (collagen type II alpha 1 chain), *Cspg4* (chondroitin sulfate proteoglycan 4), and *Otor* (otoraplin), were expressed across all samples but were more highly expressed among PDGFRα reporter^−^ periosteal cells (Fig. 7d).^31–33^ These data further confirm some of the functional differences previously observed between PDGFRα reporter^+^ and PDGFRα reporter^−^ periosteal cells, including the high osteoblastogenic potential of the PDGFRα reporter^+^ cell fraction.

The top 30 genes that were significantly upregulated and downregulated in the PDGFRα reporter^+^ group were examined (Supplementary Tables S2, S3). Among the downregulated genes, *Irx3* (Iroquois homeobox 3) and *Itga6* (integrin subunit alpha 6) are linked with chondrogenic differentiation,^34,35^ and *Cryab* (crystallin alpha B) is associated with the proliferation and extracellular matrix production of chondrocytes (Supplementary Table S2).^36^ Furthermore, among the upregulated genes, *Fermt3* (fermitin family member 3) mediates protein–protein interactions involved in integrin activation and thereby plays a role in cell adhesion and the negative regulation of chondrocyte differentiation.^37,38^ *Vegfc* (vascular endothelial growth factor C), *Wnt16*, and *Ptpn6* (protein tyrosine phosphatase nonreceptor type 6) are known to promote osteogenesis (Supplementary Table S3).^31,39,40^ Ptpn6 is a member of the protein tyrosine phosphatase family that regulates a variety of cellular processes, including cell growth and differentiation. Of these differentially expressed genes, *Fermt3* and *Ptpn6* were chosen as candidate molecules without any prior descriptions in the periosteum.

The levels of both *Fermt3* and *Ptpn6* gradually increased during the early osteogenic differentiation of mouse periosteal cells (Fig. 7e). To clarify the effects of Fermt3 and Ptpn6 on periosteal progenitor cells, we examined the consequences of the knockdown or overexpression of either gene (Fig. 7f-h). The short hairpin RNA (shRNA)-mediated knockdown or overexpression of *Fermt3* or *Ptpn6* in mouse periosteal cells was confirmed (Supplementary Fig. S15). In comparison with the results obtained with the vector control, the knockdown of either *Fermt3* or *Ptpn6* promoted the proliferation of PDGFRα reporter^+^ periosteal cells (Fig. 7f). In contrast, *Fermt3* or *Ptpn6* knockdown inhibited the osteogenic differentiation of PDGFRα reporter^+^ periosteal cells and induced a 61.2%–67.5% reduction in mineralization (Fig. 7g) and a 20.6%–43.3% reduction in osteogenic gene expression (Fig. 7h). Converse effects were observed with chondrogenic gene expression: the knockdown of either *Fermt3* or *Ptpn6* increased the expression of *Acan* and *Col2a1* (Supplementary Fig. S16a). Overall, these results suggest that the knockdown of either *Fermt3* or *Ptpn6* leads to a phenotype among PDGFRα reporter^+^ periosteal cells that is more similar to that of their PDGFRα reporter^−^ counterparts. Conversely, *Fermt3* or *Ptpn6* overexpression was observed in PDGFRα reporter^−^ periosteal cells (Fig. 7i-k). Here, *Fermt3* or *Ptpn6* overexpression reduced both PDGFRα reporter^−^ periosteal cell proliferation (Fig. 7i) and chondrogenic gene expression (Supplementary Fig. S16b) but resulted in a 73.5%–78.1% increase in bone nodule formation (Fig. 7j) and a 77.1%–192.4% increase in osteoblast-related gene expression in comparison with the results obtained with the vector control (Fig. 7k). Thus, the overexpression of either *Fermt3* or *Ptpn6* led to a ‘PDGFRα reporter^+^ periosteal cell-like’ phenotype among PDGFRα reporter^−^ periosteal cells. Moreover, forced overexpression of either ligand can improve the in vitro osteogenic differentiation potential of periosteal derivatives.

## Discussion

Although the periosteum is an inconspicuous bone-lining surface composed of cells with a fibroblastic morphology, an increasing body of evidence shows that functionally relevant cellular heterogeneity exists in this tissue compartment.^6,8,9,20,41,42^ Our study suggests that PDGFRα reporter activity marks a population of cells within the periosteum that acts as a tissue-specific stem cell reservoir for periosteal appositional bone formation and fracture repair. Moreover, PDGFRα reporter^+^ cells appear to represent the precursors of Lepr^+^/Nestin^+^/Gli1^+^ skeletal progenitor cells within the periosteum. Unlike other antigens,^10,43^ PDGFRα reporter activity appears to be a conserved marker across mouse and human bone.

Overall, the relationship between PDGFRα reporter activity and other reported periosteal progenitor cell markers needs to be further clarified. Clearly, there is some degree of overlap between other periosteal reporters and the PDGFRα reporter studied here. For example, Prx1 reporter^+^ periosteal stem cells express more PDGFRα and Periostin than Prx1 reporter^−^ cells.^41^ Periostin is essential for periosteum activation and contributes to bone repair;^41^ however, Periostin is surprisingly enriched in PDGFRα reporter^−^ cells. Ortinau et al. examined the combination of Mx1 and αSMA, which label periosteal progenitor cells that contribute to bone repair, and found that 79% of these cells are also positive for PDGFRα.^8^ Using publicly available sequencing datasets of periosteal cell derivatives, He et al. examined the transcriptome of Sox9 reporter-expressing and non-Sox9 reporter-expressing cells.^44^ Their data align with ours to some degree, which demonstrates that Sox9^-^ periosteal cells exhibit higher expression of *Pdgfrα* and *Ptpn6* but not *Fermt3*. Future studies involving a combination of PDGFRα and other periosteal stem cell markers are needed to more definitive identify the cellular hierarchy in the mouse and human periosteum.

Although not directly addressed in our study, PDGFRα is most likely both a cell marker for a periosteal stem cell population and a vital functional receptor for periosteal bone formation and fracture repair. PDGF is known to activate osteogenic differentiation and bone formation via the BMP-Smad1/5/8-Runx2/Osx axis and extracellular signal-related kinase 1/2 (ERK1/2) signaling pathway, which requires PDGFRα.^45,46^ PDGF can mobilize pericytes, some of which are skeletal stem cells, from their abluminal location on blood vessels, stimulate the expansion of these cells, and aid their organization. Furthermore, PDGF not only contributes to osteogenesis but also helps stabilize newly formed blood vessels.^47^ Thus, this factor acts to drive the multistep, multicomponent cascade of new bone formation. PDGFRα^+^ and β^+^ populations participate in fracture repair and show significant colony‐forming potential.^20^ A PDGFR-PI3K signaling axis mediates periosteal cell activation during healing.^20,48^ In addition, PDGFRβ marks reparative skeletal stem cells in the periosteal, endosteal, and perivascular niches, which altogether give rise to osteoblastic, chondrogenic, and fibroblastic progeny in the callus.^11^ The loss of PDGFRβ impairs callus formation. PDGF-PDGFRβ signaling is critical for skeletal stem cell expansion, migration, and angiotropism during bone repair.^11^ Our results show that PDGFRα reporter^+^ cells, some of which coexpress PDGFRβ,^20^ also play a vital role in bone repair.

The genes overrepresented among PDGFRα reporter^+^ periosteal progenitor cells include components of Wnt and IGF signaling. IGF1 is well known to regulate periosteal appositional bone growth,^49^ and the deletion of Wnt ligands, such as Wnt16 exert, effects on the cortical bone compartment in mice.^50^ Two relatively unknown gene candidates were further explored in the present study: Fermt3 and Ptpn6. Prior studies revealed that mutations in Fermt3 in bone marrow stromal cells resulted in enhanced chondrogenesis,^38^ but the effects on bone formation were previously unknown. In agreement with our present findings in periosteal cells, Ptpn6 promotes osteogenic differentiation and bone formation in bone marrow stromal cells.^39^ The extent to which either molecule could be used in a therapeutic context to improve bone repair has not been explored. The in vivo dysregulation of *Fermt3* and *Ptpn6* gene expression during periosteal bone formation is a logical next step.

Our study has several limitations. First, we relied primarily on an inducible reporter system, which generally correlated well but not precisely with the expression of PDGFRα protein detected by flow cytometry. This caveat should be considered in future studies in which the cell surface expression of PDGFRα is used to fractionate periosteal cell derivatives. Second, PDGFRα reporter activity is not specific to the periosteum, and we found that skeletal cells within the endosteum and trabecular bone exhibit reporter activity. Although our focus involved a single microanatomical site, the systemic DTX-mediated ablation of PDGFRα reporter^+^ cells will clearly affect more than just skeletal tissues. For example, we cannot exclude the possibility that the depletion of muscle- or marrow-resident perivascular PDGFRα reporter^+^ cells could perturb periosteal function via indirect mechanisms. Long bone mesodermal progenitors residing in skeletal muscle adjacent to a bone fracture likely play a critical role in driving the fibrotic response and fibrotic remodeling and supporting cartilage and bone formation in the fractured callus.^51^ In addition, PDGFRα^+^ cells in the bone marrow most likely play a role in fracture repair and medullary remodeling by virtue of their expression of RANKL.^52^ In our study, cell depletion showed some tissue specificity, which is an important caveat. For example, trabecular cells within the femur and even perivascular cells in nonskeletal tissues were not prominently affected with the precise DTX regimen applied (*not shown*). Third, the fracture model used in this study likely draws cell sources from multiple domains, including the periosteum, endosteum and even skeletal muscle, for repair. Pinpointing the relative cellular contributions of distinct PDGFRα reporter^+^ cellular depots would require more explicit studies using fracture models along with microdissection of various tissues. Fourth, all studies were performed with male mice. Importantly, a similar basic distribution of PDGFRα reporter activity before and after fracture was observed in both sexes. Although it is anticipated that our findings would be consistent between male and female mice, there is a notable intersection between PDGF and estrogen signaling in other tissues.^53–55^ Sex differences in periosteal cell populations would be an important area of further investigation. Fifth, some experiments utilized culture-expanded periosteal cells with or without FGF2 supplementation to improve cell growth. Clearly, some phenotypic drift in gene expression and cellular function would be anticipated with even brief culture expansion, and this fact should be considered in future work related to the studies presented herein.

In summary, PDGFRα reporter^+^ periosteal cells represent tissue-specific progenitor cells with an osteoblastogenic phenotype. The presence of PDGFRα reporter^+^ cells is vital to the established functions of the periosteum in homeostasis and repair. Newly identified genes, such as *Fermt3* and *Ptpn6*, could have future therapeutic implications in the maintenance of a healthy cortex and the prevention of fractures.

## Materials and methods

### Mice

All animal experiments were performed according to approved protocols (MO19M266, MO19M366, and MO20M142) of the Animal Care and Use Committee (ACUC) at Johns Hopkins University (JHU). The PDGFRα-CreER^TM^ animals were a kind gift from the Dwight Bergles laboratory^56^ and are commercially available (The Jackson Laboratory, Stock No. 018280, Bar Harbor, ME). Pdgfrα^mT/mG^ mice were obtained by crossing Pdgfrα-CreER^TM^ with mT/mG mice (JAX Stock No. 007576). Pdgfrα^iDTR;mT/mG^ mice were obtained by crossing Pdgfrα^mT/mG^ mice with iDTR mice (JAX Stock No. 007900). Unless otherwise specified, male mice were used in all the experiments. NOD SCID mice were purchased from the Jackson Laboratory (JAX Stock No. 001303). Tamoxifen (TM; Sigma–Aldrich, St. Louis, MO) and diphtheria toxin (DTX; Sigma–Aldrich) were injected intraperitoneally according to previously validated protocols (TM: 150 mg·kg^-1^ per day for 5 d; DTX: 45.7 μg·kg^-1^ per day for 3 d).^14,57^ TM was dissolved in sunflower seed oil (Sigma–Aldrich). Post-TM chase periods ranging from 7 d to 9 mo were assessed. When feasible, a littermate analysis was performed by investigators blinded to the mouse genotype.

### Pdgfrα cell depletion and analyses

Male 2-mo-old Pdgfrα^mT/mG^ or Pdgfrα^iDTR;mT/mG^ mice were sequentially administered TM and DTX (TM: 150 mg·kg^-1^ per day for 5 d; DTX: 45.7 μg·kg^-1^ per day for 3 d starting 10 d after TM). To confirm the efficiency of PDGFRα reporter^+^ cell depletion, mouse periosteal cells were isolated and analyzed by flow cytometry 14 d after DTX injection. To evaluate the effects on the uninjured skeleton, forelimbs and hindlimbs were analyzed 2 mo after the last DTX injection. To evaluate the effects on the injured skeleton, a closed, nonstabilized fracture model, which was adapted from previous methods, was used.^58^ Closed, nonstabilized fractures were performed in the mid-diaphysis of the right forelimbs 4 d after DTX administration. The olecranon process and the flexed carpus were placed into specially designed fixtures. Monotonic loading was performed by a displacement ramp of 0.5 mm·s^-1^ (TA Instruments, ElectroForce 3200 Series II). High-resolution Faxitron imaging was utilized to confirm fracture, and the mice were euthanized at 1 d to 1 mo after fracture. In select studies, mouse cells from the microdissected callus 7 d after fracture were digested with 1 mg·mL^-1^ collagenase P (Roche, Basel, Switzerland) and type I/II collagenase (1 mg·mL^-1^ each; Washington Biochemical, Lakewood, NJ) for 1 h at 37 °C and analyzed by flow cytometry.

### Isolation of mouse PDGFRα reporter^+^ periosteal progenitor cells

The hindlimbs were dissected, soft tissues surrounding the bones were meticulously removed, and the periosteum was left attached to the bone. Bilateral femurs and tibias were washed twice in PBS and digested with 2 mg·mL^-1^ collagenase P (Roche) in Dulbecco’s modified Eagle’s medium (DMEM) (Gibco, Grand Island, NY) containing 0.5% bovine serum albumin (Sigma–Aldrich) at 37 °C for 10 min (6 times).^42^ The digestion solution was filtered with a 40-μm filter and centrifuged. Cells from the third to sixth digestions were pooled and resuspended in αMEM (Gibco) containing 15% FBS (Gibco), 1% penicillin/streptomycin (Gibco), and 10 ng·mL^-1^ basic fibroblast growth factor (bFGF) (PeproTech, Rocky Hill, NJ). Five to eight male 9–10-week-old mice were used to yield a single cell population. The total periosteal cells obtained either immediately after digestion or after one passage were subjected to FACS and flow cytometry analysis. The cells were incubated with anti-CD31-allophycocyanin (1:30), anti-CD45-allophycocyanin (1:30), and anti-Ter119-allophycocyanin (1:30) antibodies for 20 min on ice (antibody are provided details in Supplementary Table S4). For the assessment of cell viability, propidium iodide (BD Pharmingen, San Diego, CA) was added. FACS was then performed using Beckman MoFlo (Beckman, Indianapolis, IN), and the data were analyzed using Summit Software (PrismHR, Hopkinton, MA). Gating strategies were performed to isolate either PDGFRα reporter^−^ (CD31^−^CD45^−^Ter119^−^mTdtomato^+^) or PDGFRα reporter^+^ (CD31^−^CD45^−^Ter119^−^mGFP^+^) cells. The sorted cells were cultured with αMEM, 15% FBS, and 1% penicillin/streptomycin with or without 10 ng·mL^-1^ mouse bFGF. The medium was changed every 3 d unless otherwise noted.

### Isolation of human PDGFRα^+^ periosteal progenitor cells

Human periosteum was obtained from adult patient donors under IRB approval at Johns Hopkins University with a waiver of informed consent. All specimens were collected from the long bones (femur or tibia) resected for a nonneoplastic medical indication and were otherwise deidentified. The periosteum was detached from the underlying bone using a periosteal elevator and then finely minced using a scalpel and dissecting scissors. The tissue was digested with 2 mg·mL^-1^ collagenase type II in DMEM for 90 min under agitation at 37 °C. Undigested tissue was sequentially filtered through 100-μm and 40-μm filters. The cells were separated by centrifugation and resuspended in red blood cell lysis buffer (155 mmol·L^-1^ NH_4_Cl, 10 mmol·L^-1^ KHCO3, and 0.1 mmol·L^-1^ EDTA) for 10 min at room temperature (RT). Trypan blue staining was performed to confirm the cell viability. After centrifugation, the periosteal cells were resuspended in HBSS containing 0.5% bovine serum albumin. PDGFRα^−^ and PDGFRα^+^ periosteal cells were isolated via FACS using a mixture of the following directly conjugated antibodies for 20 min on ice: anti-CD31-allophycocyanin-Cy7 (1:100), anti-CD45-allophycocyanin-Cy7 (1:30), and anti-CD140a-phycoerythrin (1:5) (antibody details are provided in Supplementary Table S4). The solution was passed through a 40-μm filter and then run on a Beckman MoFlo (Beckman, Indianapolis, IN). Gating strategies were utilized to isolate either PDGFRα^−^CD31^−^CD45^−^ or PDGFRα^+^CD31^−^CD45^−^ cells. The sorted cells were cultured at 37 °C in a humidified atmosphere containing 5% CO_2_. All cells were cultured in αMEM, 15% FBS, 1% penicillin/streptomycin, and 2 ng·mL^-1^ bFGF. The medium was changed every 3 d unless otherwise noted.

### Proliferation, osteogenic/chondrogenic differentiation, and qRT–PCR

Cells (2 × 10^3^) were cultured in 96-well plates, and their proliferation was measured after 72 h of culture in growth medium using the CellTiter96^®^ Aqueous One Solution Cell Proliferation Assay kit (MTS, G358A; Promega, Madison, WI). Briefly, 20 μL of MTS solution was added to each well, and the plate was incubated for 1 h at 37 °C. The absorbance was measured at 490 nm using an Epoch microspectrophotometer (Bio-Tek, Winooski, VT). Osteogenic differentiation experiments using medium with or without bFGF supplementation during the expansion period were repeated. Osteogenic differentiation was performed using osteogenic differentiation medium consisting of αMEM, 10% FBS, 1% penicillin/streptomycin, 100 nmol·L^-1^ dexamethasone, 50 μmol·L^-1^ ascorbic acid, and 10 mmol·L^-1^ β-glycerophosphate (Sigma–Aldrich). The medium was changed every 3 d. After 7 d of differentiation, the cells were harvested, and the expression of osteogenic marker genes was detected. After 8–16 d of differentiation, the cultures were stained with alizarin red S (Sigma–Aldrich) to detect mineralization. Calcium precipitate was quantified by detecting the absorbance at 548 nm after dissolving with 0.1 mol·L^-1^ sodium hydroxide. For chondrogenic differentiation, the cells were seeded in a high-density micromass environment (1 × 10^6^ cells in 10 μL of medium/drop) in 12-well plates and cultured with chondrogenic differentiation medium (αMEM, 1% penicillin/streptomycin, 10% FBS with 10 ng·mL^-1^ transforming growth factor-β3 (R&D Systems, Minneapolis, MN), 100x ITS + Premix (Corning Incorporated, Corning, NY), 100 μg·mL^-1^ pyruvate, 40 μg·mL^-1^ proline, 50 μg·mL^-1^ ascorbic acid, and 100 nmol·L^-1^ dexamethasone (Sigma–Aldrich)). The medium was changed every 3 d. After 7 d of differentiation, the cells were harvested, and the expression of chondrogenic marker genes was detected. After 21 d of differentiation, micromass cultures were cryosectioned at 18-μm thickness and stained with toluidine blue solution or safranin O-fast green. Gene expression analysis was conducted by quantitative real-time polymerase chain reaction (qRT–PCR). TRIzol (Life Technologies Corporation, Gaithersburg, MD) was used for total RNA isolation. Subsequently, cDNA synthesis was performed using an iScript cDNA Synthesis Kit (Bio–Rad, Hercules, CA) following the manufacturer’s instructions. Real-time PCR was performed using SYBR™ Green PCR Master Mix (Life Technology), and detection was performed with a QuantStudio 5 Real-Time PCR system instrument (Thermo Scientific, Waltham, MA). GAPDH was used as an internal control for all genes. Primer information is provided in Supplementary Table S5.

### Cell implantation

Mouse or human PDGFRα cell subsets were assessed for ectopic bone formation. Mouse PDGFRα reporter^−^ or reporter^+^ cells (3.0 × 10^6^) were mixed with 45 mg of hydroxyapatite/β-tricalcium phosphate (HA/β-TCP) mixture (w/w = 4:6, Zimmer Dental Inc., Carlsbad, CA). After anesthesia and analgesia, subcutaneous implants were placed on the dorsal surface of 8‐week‐old male NOD SCID mice (Stock # 001303, The Jackson Laboratory; 2 implants per mouse). The samples were analyzed after 8 weeks. An outline of the animals in each experimental group is provided in Supplementary Table S6.

Human PDGFRα^−^ or PDGFRα^+^ cells (3.0 × 10^6^) derived from the same human periosteum sample were resuspended in 40 μL of PBS and mechanically mixed with 50 mg of demineralized bone matrix (DBX) putty (morselized human cortical bone in sodium hyaluronate with a 31% bone content by weight, courtesy of Musculoskeletal Transplant Foundation, Edison, NJ). After anesthesia and analgesia, pockets were cut into the biceps femoris muscles, and cells mixed with DBX were intramuscularly implanted into the thigh muscle pouch of 8-week-old male NOD SCID mice. The muscle and skin were closed with 4–0 Vicryl*Plus sutures (Ethicon Endo-Surgery, Blue Ash, OH). An outline of the animals in each experimental group is presented in Supplementary Table S7.

### Radiographic analyses

Samples were scanned using a high-resolution micro-CT imaging system (SkyScan 1275; Bruker MicroCT N.V., Kontich, Belgium) at an image resolution of 10–15 μm. The scanner was set to the following parameters: aluminum filter of 1 mm, X-ray voltage of 65 kVP, anode current of 153 μA, exposure time of 160–218 ms, frame averaging of 6, and rotation step of 0.3 degrees. Three-dimensional images were then reconstructed using image reconstruction software (NRecon, v1.7.0.4, SkyScan, Bruker). For 3D morphometric analyses of the images, CTAn (v1.16, SkyScan, Bruker), CTVox (v3.2, SkyScan, Bruker), and CTVol (v2.0, SkyScan, Bruker) software were used. Cross-sectional images of the femur and ulna were obtained to perform 2D morphometric analysis of cortical bone and 3-dimensional histomorphometric analysis of new bone. For analysis of the fractured callus, the region of interest (ROI) was delineated into 0.5-mm segments with a threshold value of 65–255, encompassed the entire callus and excluded native bony elements. For the cortical bone analysis, the ROI was delineated into 0.75-mm segments around the midpoint of the femur or ulna with a threshold value of 110–255. For subcutaneous bone formation analysis, the ROI was set to a rectangle (2.2 mm × 1.1 mm), the analysis thickness was 0.66 mm, and the threshold value was 70–110. To remove the influence of HA/TCP, all microCT calculations were performed by subtracting the mean values of BV and BV/TV obtained from the control group (HA/TCP alone). For intramuscular implantation analysis, the ROI was set to a rectangle (1.5 mm × 0.75 mm), the analysis thickness was 0.45 mm, and the threshold value was 100–255. All analyses were performed in a blinded manner.

### Histologic and immunohistochemical analyses

Tissues were fixed in 4% paraformaldehyde (PFA) for 24 h, decalcified in 14% EDTA for 60–90 d, and embedded in optimal cutting temperature compound (OCT) (Sakura, Torrance, CA). The samples were cryo-sectioned at 20-μm thickness. The histochemical staining included routine H&E or alkaline phosphatase (ALP) staining. ALP staining was performed according to the manufacturer’s instructions (Sigma–Aldrich). For immunofluorescent staining, all sections were incubated with trypsin enzymatic antigen retrieval solution (Abcam, Cambridge, MA, USA) for 5 min at RT and blocked with either 5% goat serum or 5% donkey serum in PBS for 1 h at RT. The primary antibodies were used for either overnight incubation at 4 °C or incubation at 25 °C for 3 h (antibody details are provided in Supplementary Table S4). Nonimmune immunoglobulin of the same isotype as the primary antibodies was used as a negative control. Subsequently, anti-mouse Alexa Fluor® 647-conjugated, anti-rabbit Alexa Fluor® 647-conjugated, anti-chicken Alexa Fluor® 647-conjugated, or anti-biotin Alexa Fluor® 647-conjugated secondary antibodies (1:200) were used for incubation for 2 h at RT. For the visualization of reporter activity, a wild-type specimen prepared in the same manner was used as a control to identify the autofluorescence intensity. DAPI mounting medium (H-1500, Vector laboratories, Burlingame, CA) was used. All histological sections were examined under a Zeiss 800 confocal microscope (Zeiss, Thornwood, NY) or Leica DM6 microscope (Leica Microsystems Inc, Wetzlar, Germany).

### Transcriptomics

Periosteal cells were isolated using the above-described method and briefly cultured by propagation in αMEM, 15% FBS, and 1% penicillin/streptomycin with 10 ng·mL^−1^ mouse bFGF. After one passage, PDGFRα reporter^−^CD31^−^CD45^−^Ter119^−^ and PDGFRα reporter^+^CD31^−^CD45^−^Ter119^−^ mouse periosteal cells were identified by FACS, and global gene expression analysis was immediately performed by total RNA sequencing. Briefly, total RNA was extracted from PDGFRα reporter^−^ and PDGFRα reporter^+^ cells using TRIzol (Life Technologies Corporation). Gene expression analyses were performed through deep sequencing with the Illumina NextSeq 500 platform (Illumina, San Diego, CA) by the JHMI Transcriptomics and Deep Sequencing Core. The data analyses were performed using various software packages, including Partek Genomics Suite, Spotfire DecisopnSite with Functional Genomics, and QIAGEN Ingenuity® Pathway Analysis.

### Knockdown

In select experiments, the shRNA-mediated knockdown of *Fermt3* or *Ptpn6* was performed in primary mouse PDGFRα reporter^+^ periosteal cells. *Fermt3* and *Ptpn6* shRNA were obtained from Sigma–Aldrich, and the pLKO.1 vector was obtained from Addgene (Watertown, MA). The shRNA was transfected using TransIT^®^-LT1 Transfection Reagent (Mirus Bio, Madison, WI) as described by the manufacturer. The medium was changed after 4 h and replaced by osteogenic medium after 24 h. After 7 d of differentiation, osteogenic marker genes were detected by qRT–PCR. The cultures were stained with alizarin red S to detect mineralization after 11 d of differentiation, and cellular proliferation was measured after 48 h of culture in growth medium as described above.

### Overexpression

*Fermt3* or *Ptpn6* overexpression in primary PDGFRα reporter^−^ periosteal cells was performed. The pCMV6-AC-GFP vector and *Fermt3*/*Ptpn6* plasmids were obtained from Origene (Watertown, MA). The plasmid was transfected using TransIT^®^-LT1 Transfection Reagent (Mirus Bio, Madison, WI) as described by the manufacturer. The medium was changed after 4 h and replaced by osteogenic medium after 24 h. After 7 d of differentiation, osteogenic marker genes were detected by qRT–PCR. The cultures were stained with alizarin red S to detect mineralization after 8 d of differentiation, and cellular proliferation was measured after 48 h of culture in growth medium as described above.

### Statistical analysis

The data are presented as the means ± one-SD values. The statistical analyses were performed using GraphPad Prism (Version 7.0). The in vitro experiments were performed in biological and experimental triplicates. The number of animals used in the in vivo experiments is shown in the figure legends and in Supplementary Tables S6 and S7. For the cell depletion experiments, our depletion efficiency resulted in effect sizes of at least 2.38. For cell implantation, our initial in vitro studies comparing PDGFRα reporter^−^ and PDGFRα reporter^+^ cells resulted in effect sizes of at least 2.73. For these scenarios and using 4 mice per group, a two-sample t test would provide 80% power to detect effect sizes of at least 2.15 based on the assumption of a two-sided 0.05 level of significance. The Kolmogorov–Smirnov test was used to confirm the normal distribution of the data. A two-tailed Student’s *t* test was used for two-group comparisons. One-way or two-way ANOVA followed by Tukey’s multiple-comparisons test was used for comparisons of 3 or more groups. *P < 0.05, **P < 0.01, and ***P < 0.001 were considered to indicate significance.

## Supplementary information

Supplementary Information

Supplementary Information clean

## Data Availability

Expression data that support the findings of this study have been deposited in the Gene Expression Omnibus (GEO) under the accession code GSE154003.
