# Supplementary material for: PDGFRα reporter activity identifies periosteal progenitor cells critical for bone formation and fracture repair
Source: Bone Res. 2022 Jan 25;10:7. doi: 10.1038/s41413-021-00176-8 (PMC8786977; doi:10.1038/s41413-021-00176-8)
Supplement: Supplementary file 1 — Supplementary Information [file 41413_2021_176_MOESM1_ESM.docx]

**Supplementary Information**


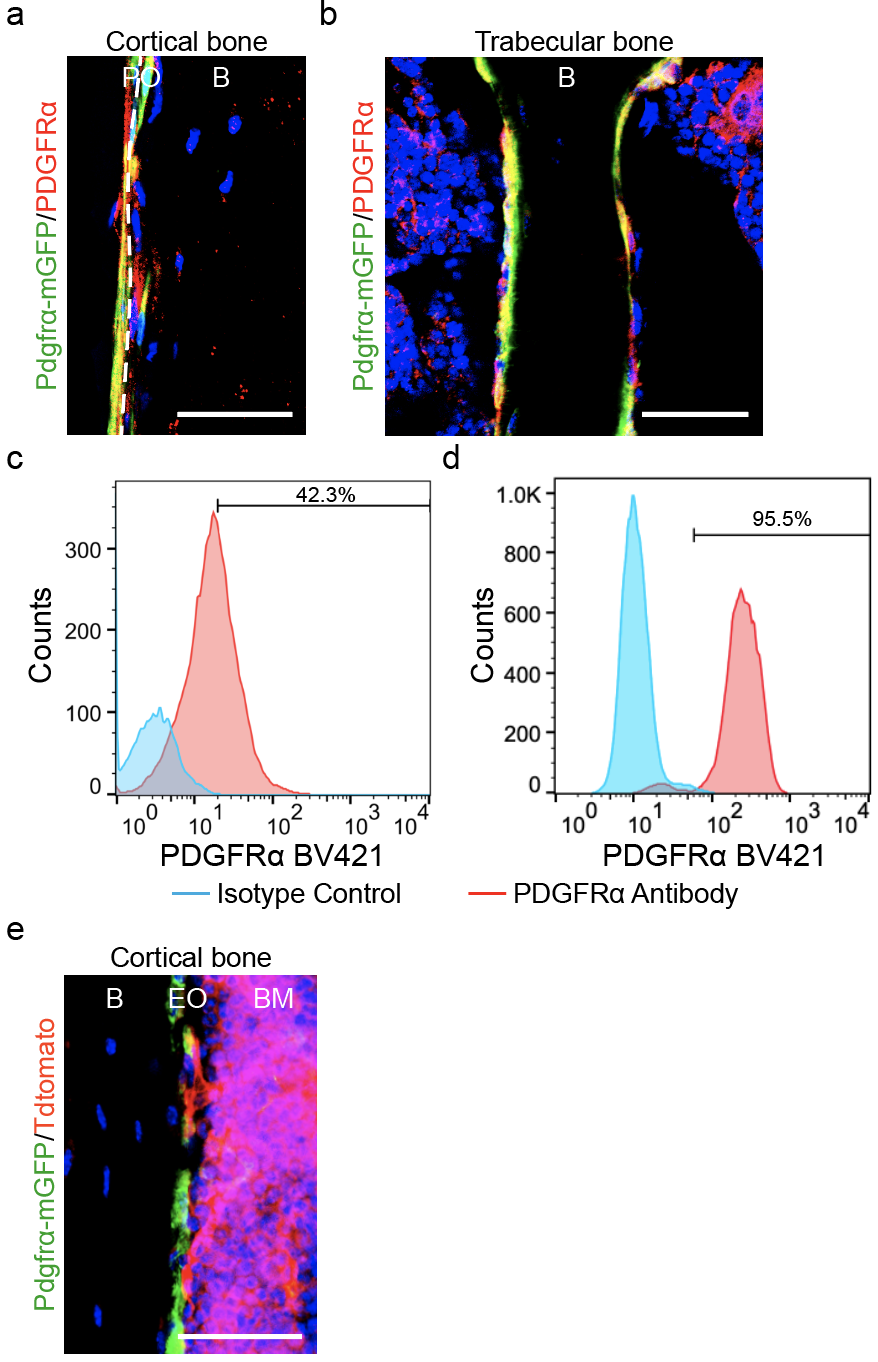


**Supplementary Figure S1. PDGFRα-CreER reporter activity location and correlation with PDGFRα expression. (a,b)** Immunohistochemical stains for PDGFRα within **(a)** femoral diaphyseal periosteum and **(b)** trabecular bone of the proximal femur, using Pdgfrα^mT/mG^ reporter sections. GFP indicates reporter activity, while Tdtomato expression is not shown. Dashed line indicates the limit between the inner and outer layers of periosteum. N=3 male 2 mo old animals, analyzed 14 d after TM administration. **(c,d)** GFP^+^CD45^-^CD31^-^CD119^-^ cells isolated from microdissected mouse periosteum of Pdgfrα^mT/mG^ animals were analyzed by flow cytometry for PDGFRα expression in comparison to isotype control. Histograms showed the percentage of PDGFRα^+^ cells in **(c)** freshly isolated cells 7 d after TM or **(d)** the same cells after culture expansion (Passage 4). **(e)** PDGFRα reporter^+^ (GFP^+^) activity within the femoral diaphyseal endosteum. Cells derived from male 10 wk old animals. Scale bars: 50 μm. B, bone; BM, bone marrow; EO, endosteum; PO, periosteum.


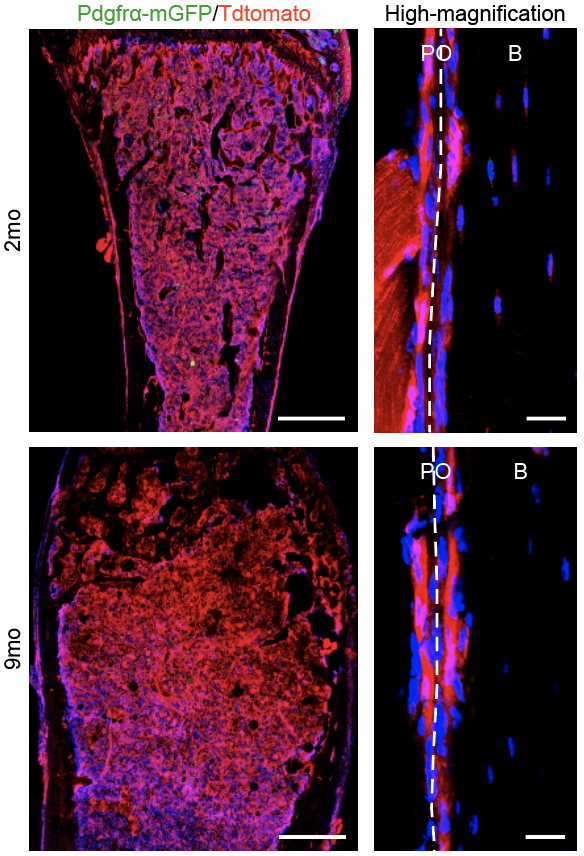


**Supplementary Figure S2. Minimal PDGFRα reporter activity without tamoxifen.** mGFP and Tdtomato are visualized in the proximal femur of 2 and 9 mo old Pdgfrα^mT/mG^ male reporter animals, shown as tile scans and high magnification images of the periosteum. Dashed lines indicate the limit between the inner and outer layers of periosteum. Scale bar: 500 μm (left panel) and 20 μm (right panel). PO, periosteum; B, bone.

**
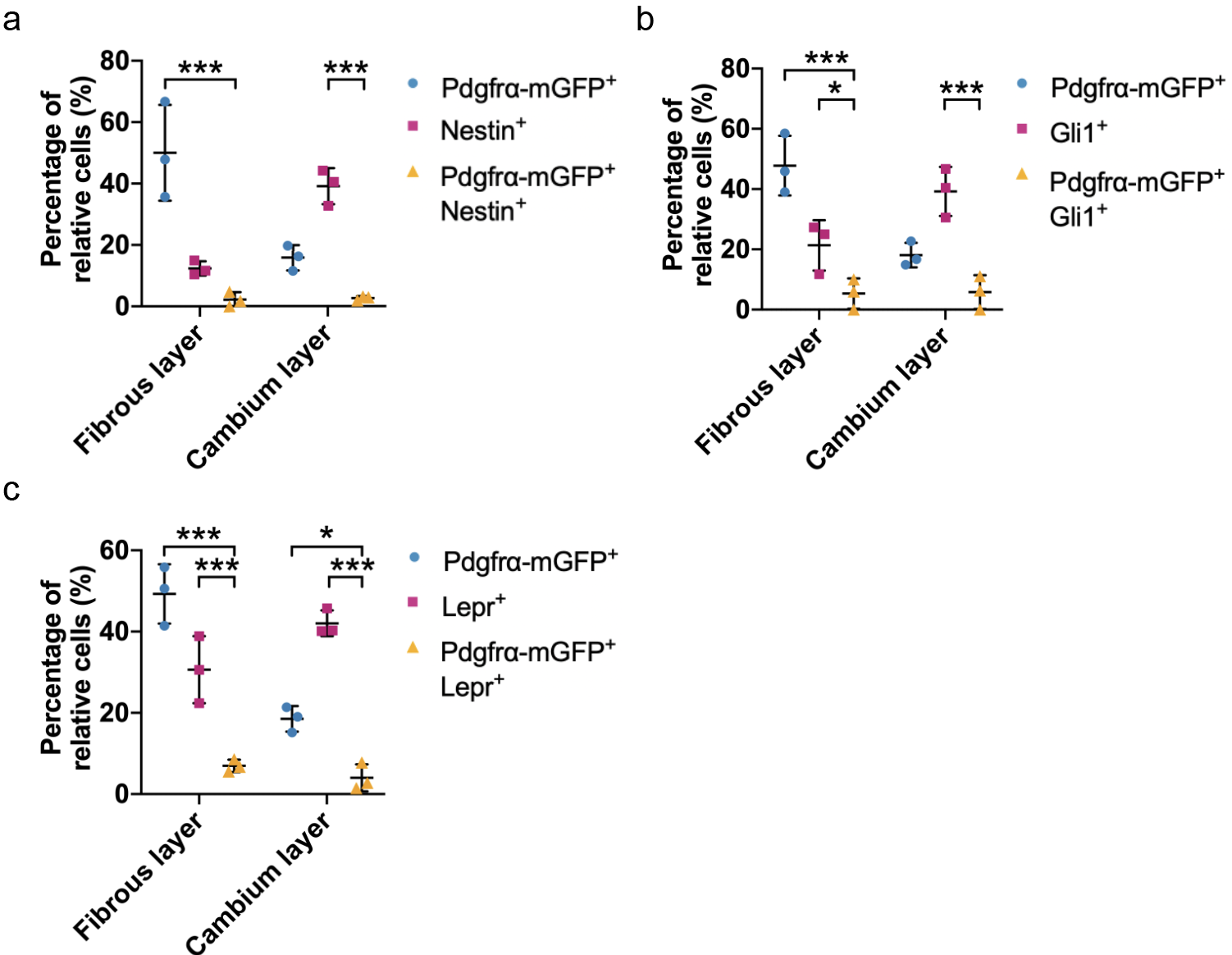
**

**Supplementary Figure S3. Quantification of periosteal PDGFRα reporter^+^ cells in the fibrous and cambium layers of periosteum.** The frequency of PDGFRα reporter expression in fibrous and cambium layers of diaphyseal periosteum was examined alone or in combination with immunohistochemical detection of Nestin, Gli1, or Lepr (Leptin receptor). Analysis was performed within the femoral periosteum of Pdgfrα^mT/mG^ reporter animals. Each quantification is reported as the percentage of single positive cells or double positive cells in each tissue layer. The corresponding images can be found in **Fig. 1e**. **(a)** Percentage of PDGFRα reporter^+^, Nestin^+^, or PDGFRα reporter^+^Nestin^+^ cells among fibrous or cambium layers. **(b)** Percentage of PDGFRα reporter^+^, Gli1^+^, or PDGFRα reporter^+^Gli1^+^ cells among fibrous or cambium layers. **(c)** Percentage of PDGFRα reporter^+^, Lepr^+^, or PDGFRα reporter^+^Lepr^+^ cells among fibrous or cambium layers. Dot plots represent an individual animal, while whisker plots indicate mean values and one standard deviation. Quantification based on N=3 male 2 mo old animals, with a 2 wk TM chase period. **P*<0.05; ***P*<0.01; ****P*<0.001 as assessed using two-way ANOVA with Tukey’s multiple comparisons test.

**
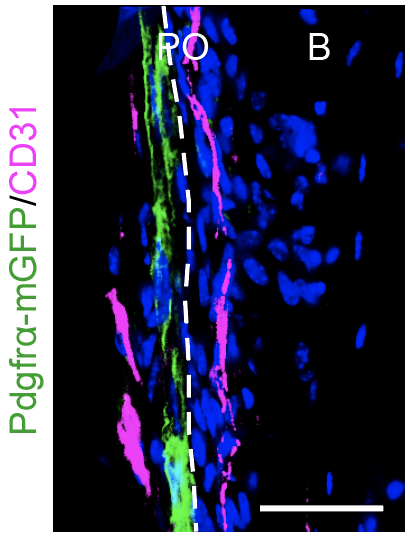
**

**Supplementary Figure S4. PDGFRα reporter^+^ periosteal cells do not principally reside in a perivascular niche.** Immunohistochemical stain for CD31 within femoral periosteum using Pdgfrα^mT/mG^ reporter sections. Pdgfrα^mT/mG^ animals (N=3, male, 2 mo old) were administered TM, and reporter activity was examined 2 wks later. Tdtomato is not shown. Dashed line indicates the limit between the inner and outer layers of periosteum. Scale bar: 50 μm. PO, periosteum; B, bone.


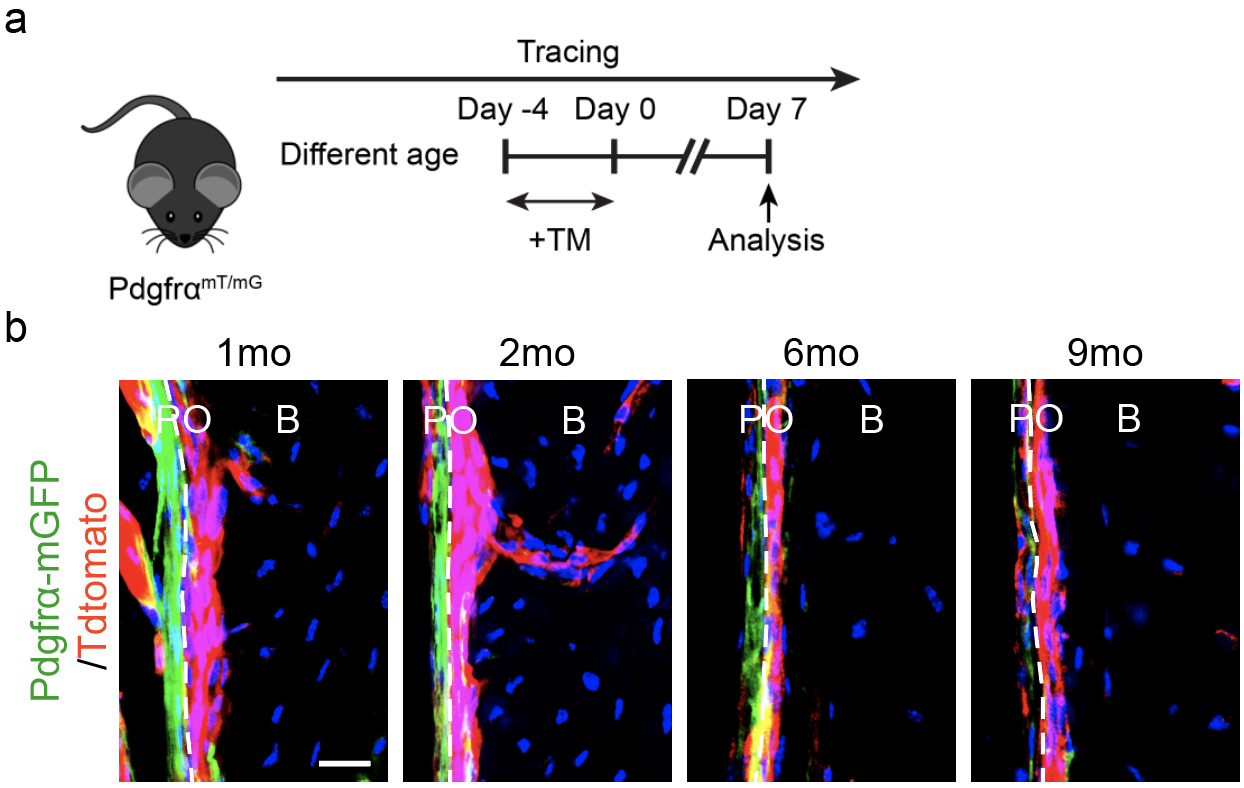


**Supplementary Figure S5. PDGFRα reporter^+^ periosteal progenitor cells reduce with age. (a)** Male Pdgfrα^mT/mG^ animals were administered tamoxifen (TM) at 1, 2, 6, or 9 months of age. In each case, the location of Pdgfrα-labeled cells was examined at 7 d after TM administration. **(b)** Representative images of femoral diaphyseal periosteum from Pdgfrα^mT/mG^ mice at different ages after TM administration. Scale bar: 20 μm. Nuclei, DAPI (4′,6-diamidino-2-phenylindole, blue). PO, periosteum; B, bone. Dashed lines in **b** indicate the limit between the inner and outer layers of periosteum. N=3-4 animals per timepoint.


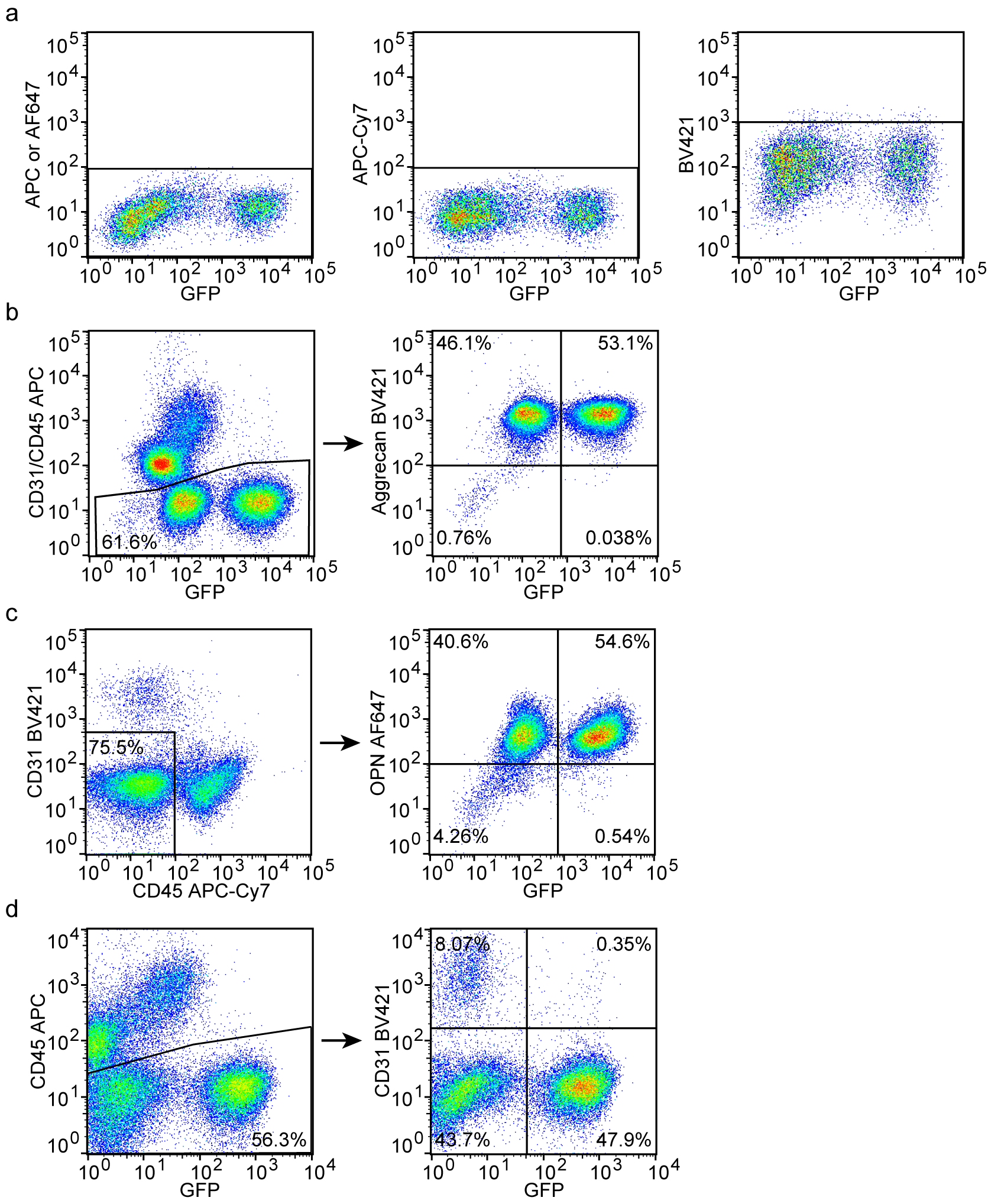


**Supplementary Figure S6. PDGFRα reporter^+^ cells give rise to osteochondral cells, but not endothelial cells after fracture.** Fracture calluses from Pdgfrα^mT/mG^ animals (male, 2-month-old) were microdissected, dissociated and analyzed by flow cytometry 7 d after injury. **(a)** Isotype control was shown to set the negative gates. **(b)** Analysis of GFP within Aggrecan^+^ cells among freshly isolated CD31^-^CD45^-^ periosteal cells. **(c)** Analysis of GFP expression within osteopontin (OPN)^+^ cells among freshly isolated CD31^-^CD45^-^ periosteal cells. **(d)** Analysis of GFP expression within CD31^+^ cells among freshly isolated CD45^-^ periosteal cells. The total number of events is 12,130, 50,175, 68,830, and 69,792 in a-d, respectively.


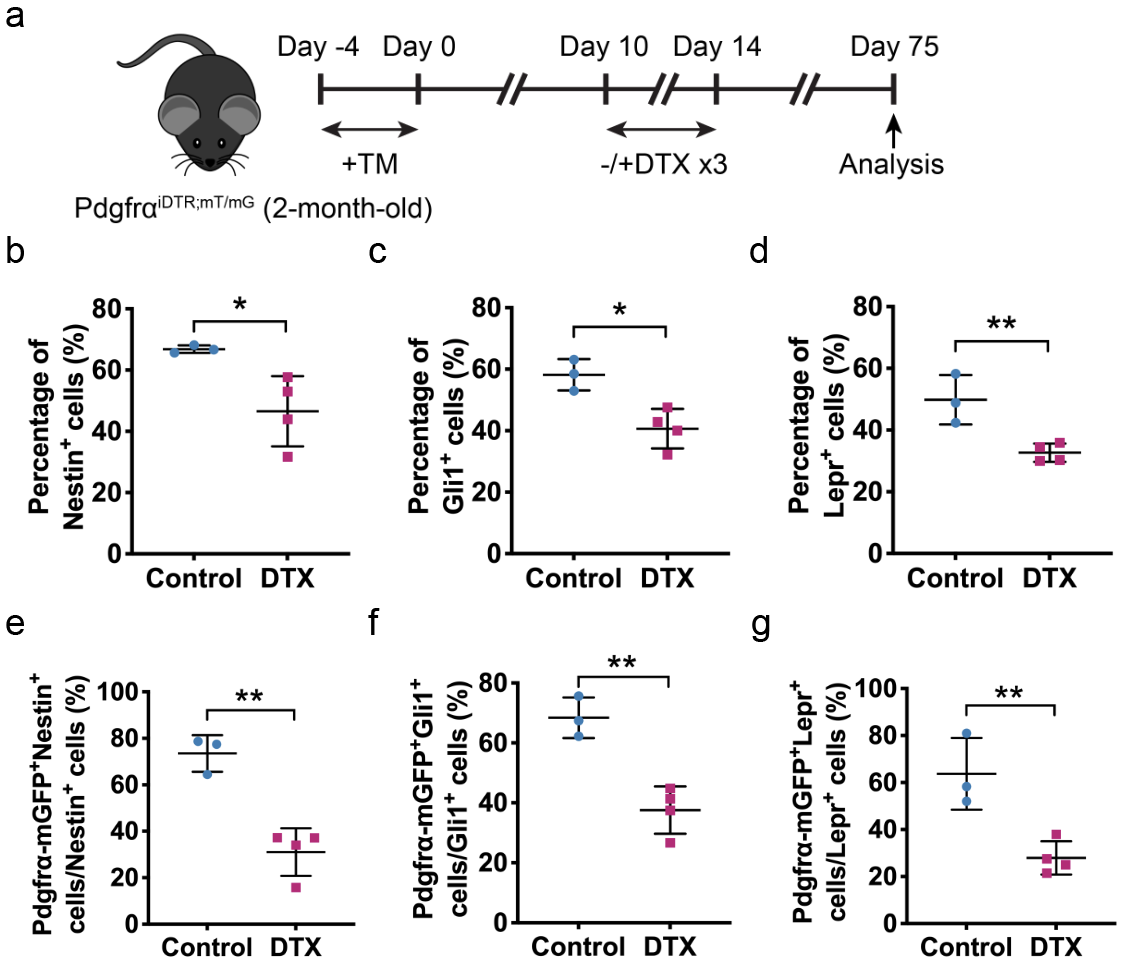


**Supplementary Figure S7. Ablation of PDGFRα reporter^+^ cells decreases skeletal progenitor cell frequency in periosteum. (a)** Pdgfrα^iDTR;mT/mG^ animals (male, 2-month-old) were sequentially administered tamoxifen (TM) followed by diphtheria toxin (DTX), with analysis performed after 2 mo. **(b-d)** The percentage of Nestin^+^ **(b)**, Gli1^+^ **(c)**, and Lepr (Leptin receptor)^+^ **(d)** cells in periosteum, as assessed by immunohistochemistry. **(e-g)** Percentage of PDGFRα reporter^+^Nestin^+^ **(e)**, PDGFRα reporter^+^Gli1^+^ **(f)**, and PDGFRα reporter^+^Lepr^+^ **(g)** cells in the diaphyseal periosteum. The corresponding images can be found in **Fig. 3d**. DTX: diphtheria toxin. Dot plots represent an individual animal, while whisker plots indicate mean values and one standard deviation. N=3-4 animals per group. **P*<0.05; ***P*<0.01 as assessed using two-tailed Student’s *t*-tests.


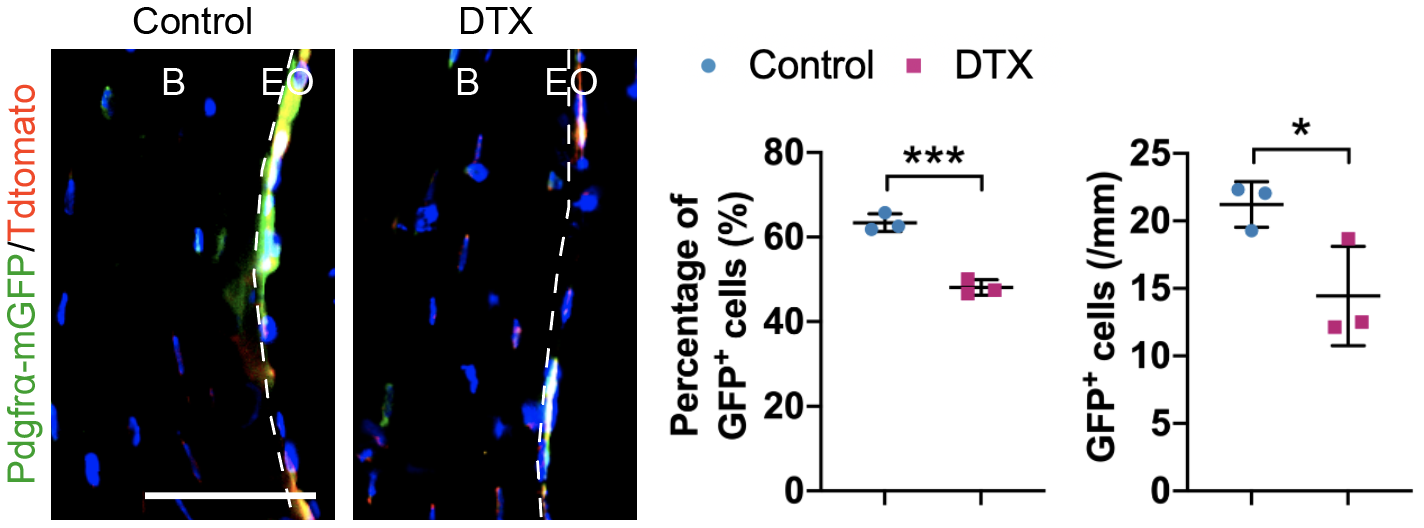


**Supplementary Figure S8.** **PDGFRα reporter activity (GFP) in endosteum was reduced among DTX-treated animals.** Pdgfrα^iDTR;mT/mG^ animals (male, 2 mo old) were sequentially administered tamoxifen (TM) and diphtheria toxin (DTX) with analysis performed after 2 mo. Representative images of PDGFRα reporter activity^+^ (GFP^+^) endosteal osteoblasts among DTX- or control-treated Pdgfrα^iDTR;mT/mG^ animals. Nuclei, DAPI (4′,6-diamidino-2-phenylindole, blue). Percentage of mGFP^+^ endosteal osteoblasts / total endosteal osteoblasts and the number of mGFP^+^ endosteal osteoblasts/mm were shown. Scale bars: 50 μm. N=3 animals per group. Dot plots represent an individual animal, while whisker plots indicate mean values and one standard deviation. **P*<0.05; ****P*<0.001 as assessed using two-tailed Student’s *t*-tests.


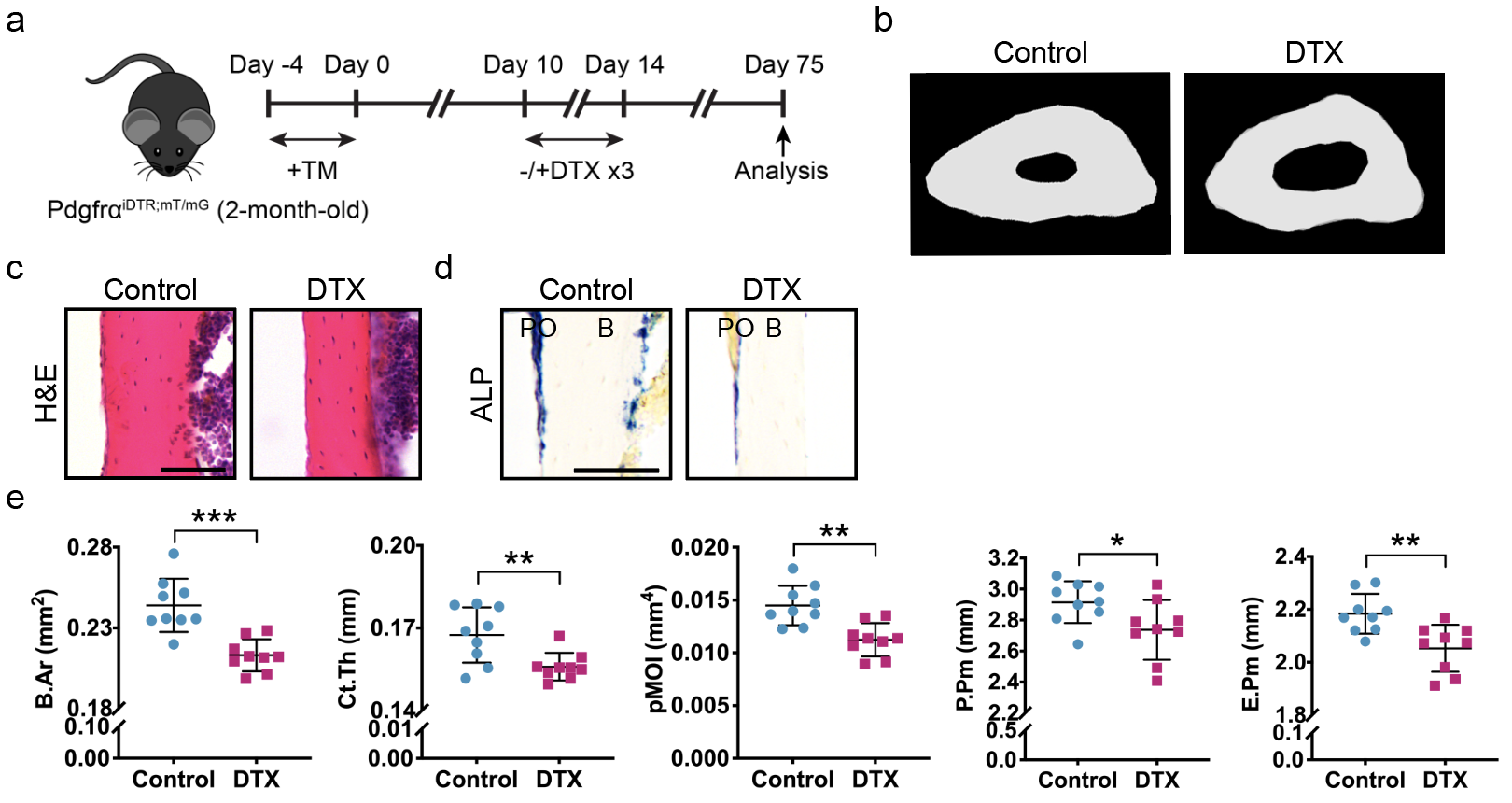


**Supplementary Figure S9.** **Ablation of PDGFRα^+^ cells reduced the cortical bone mass of ulna. (a)** Male 2-month-old Pdgfrα^iDTR;mT/mG^ animals were administered tamoxifen (TM) followed by diphtheria toxin (DTX). Cortical bone mass in the midshaft ulna was measured 2 mo after DTX. **(b)** Micro-CT reconstructions of cortical bone in the midshaft ulna. **(c,d)** Representative histologic appearance by **(c)** H&E and **(d)** alkaline phosphatase (ALP) staining. **(e)** Quantitative analysis of micro-CT images among the control and DTX groups, including bone area (B.Ar), cortical thickness (Ct.Th), polar moment of inertia (pMOI), periosteal perimeter (P.Pm), and endosteal perimeter (E.Pm). Scale bar: 50 μm. PO, periosteum; B, bone. In graphs, dot plots represent an individual animal, while whisker plots indicate mean values and one standard deviation. N=9 per group animals. **P*<0.05; ***P*<0.01; ****P*<0.001 as assessed using two-tailed Student’s *t*-tests.


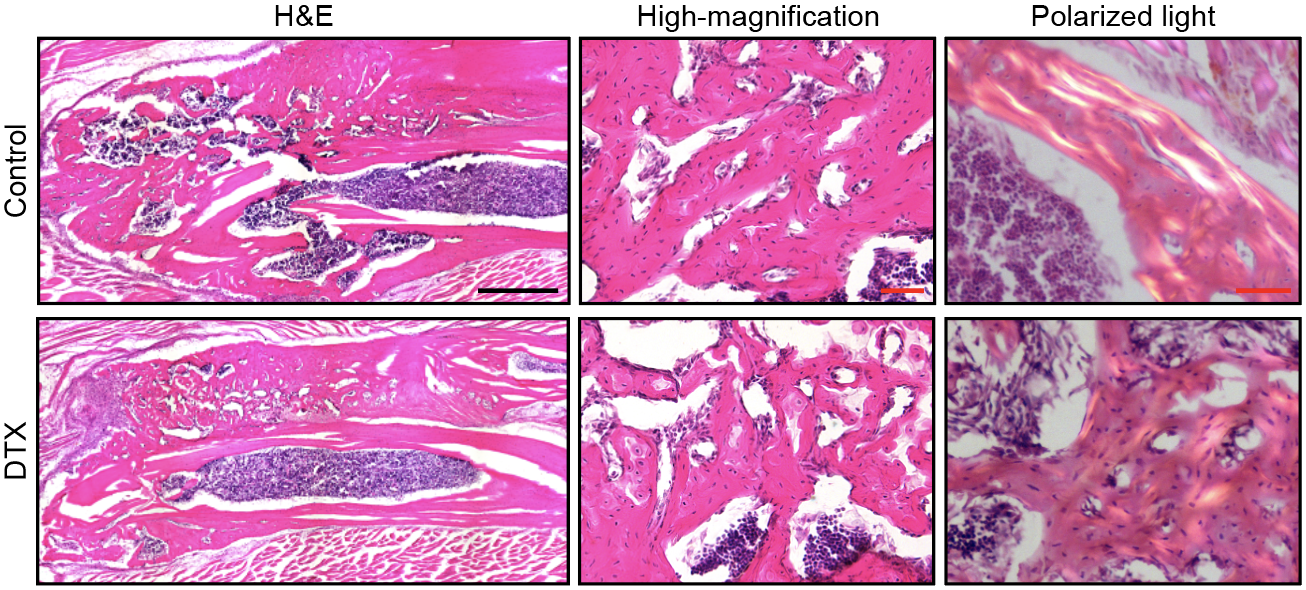


**Supplementary Figure S10. Representative H&E-stained images of the fracture callus in Pdgfrα^iDTR;mT/mG^ animals, 30 d after fracture.** Images shown at low and high magnification, and under polarized light. Black scale bars: 500 μm. Red scale bars: 50 μm. N=4 animals per group.


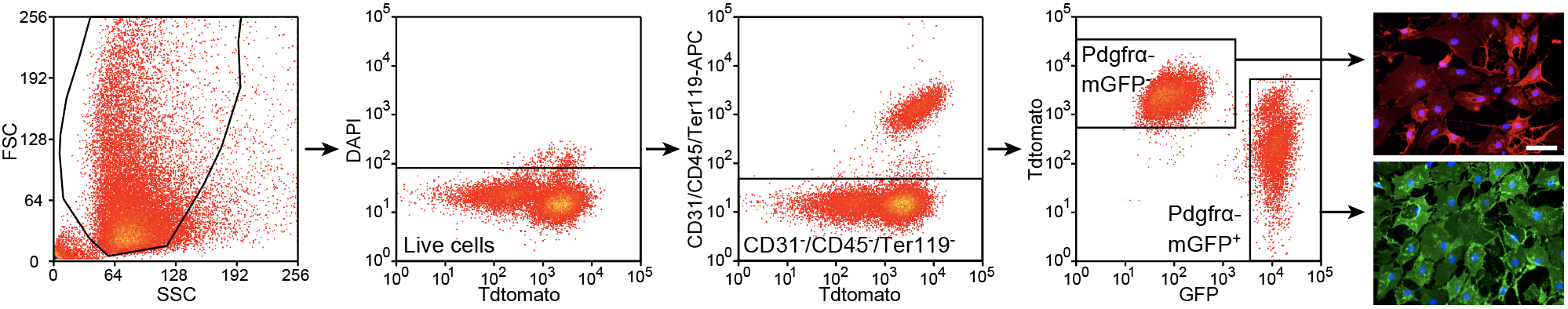


**Supplementary Figure S11. Isolation methods of PDGFRα reporter^+^ periosteal progenitor cells.** Mouse periosteal cells are derived by size distribution, followed by exclusion of DAPI^+^ cells, followed by exclusion of CD31, CD45, and Ter119 expressing cells. Among CD31^-^CD45^-^Ter119^-^ periosteal cells, PDGFRα reporter^-^ (mTdtomato^+^, red) and PDGFRα reporter^+^ (mGFP^+^, green) cell isolation was performed from Pdgfrα^mT/mG^ mouse periosteum. Isolation was performed 2 wks after TM from microdissected mouse hindlimbs (femur/tibia). Scale bar: 50 μm.


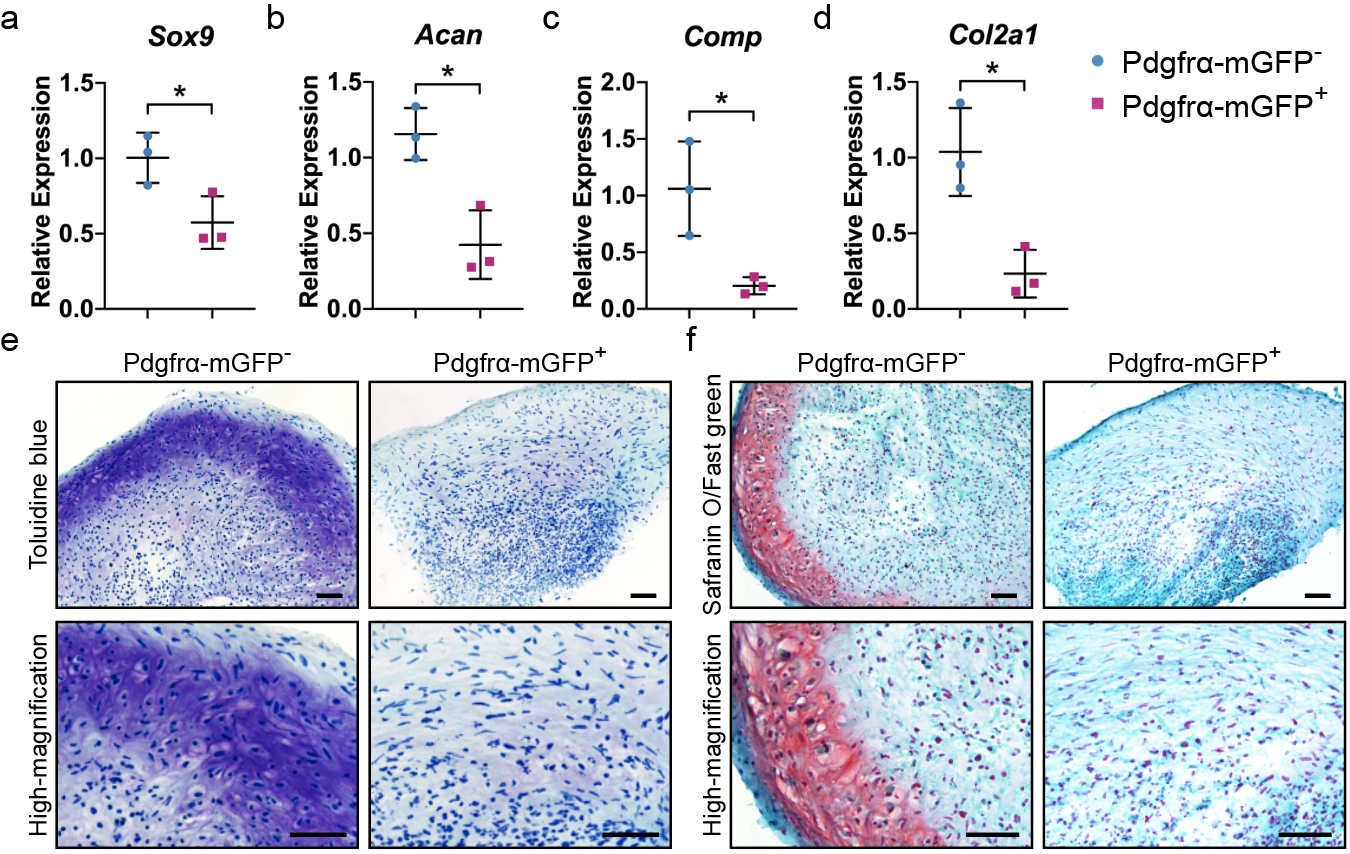


**Supplementary Figure S12. Chondrogenic differentiation of PDGFRα reporter^-/+^ cells.** Chondrogenic gene expression among PDGFRα reporter^-/+^ mouse periosteal cells, including **(a)** *SRY-Box transcription factor 9* (*Sox9*), **(b)** *Aggrecan* (*Acan*), **(c)** *Cartilage oligomeric matrix protein* (*Comp*), and **(d)** *Collagen type 2 a1* (*Col2a1*) at 7 d of differentiation in micromass culture. **(e,f)** Chondrogenic differentiation, as assessed by **(e)** Toluidine blue/fast green and **(f)** Safranin o/fast green staining at 21 d of differentiation in high density micromass culture. Cells were defined by FACS as PDGFRα reporter^-^(Tdtomato^+^)CD31^-^CD45^-^Ter119^-^ and PDGFRα reporter^+^(GFP^+^)CD31^-^CD45^-^Ter119^-^ populations from microdissected mouse hindlimbs of Pdgfrα^mT/mG^ animals, and chondrogenic differentiation assays were performed on passage 5 cells. Experiments were performed in experimental triplicate. Scale bar: 50 μm. Dot plots represent an individual sample, while whisker plots indicate mean values and one standard deviation. **P*<0.05 as assessed using two-tailed Student’s *t*-tests.


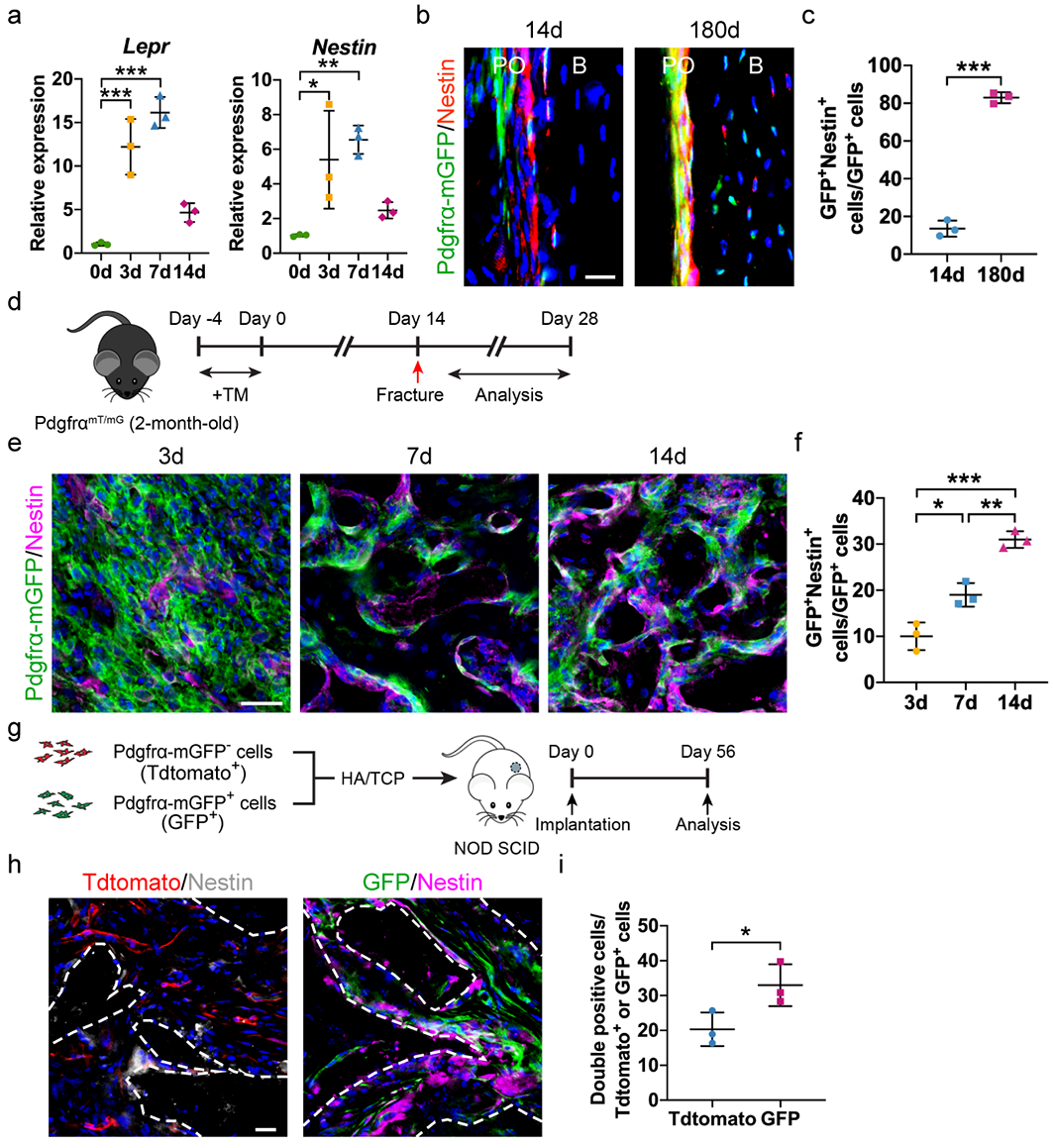


**Supplementary Figure S13. PDGFRα reporter^+^ periosteal progenitors give rise to Nestin^+^ PDGFRα reporter^+^ periosteal cells during bone formation. (a)** *In vitro* expression of *Nestin* (*Nes*) and *leptin receptor* (*Lepr*) during osteogenic differentiation. Gene expression was examined using mouse PDGFRα reporter^+^ periosteal cells from d 0-14 of osteogenic differentiation, as assessed by qPCR. **(b)** Nestin expression among the uninjured femoral diaphyseal periosteum after long term chase periods (samples also shown in **Fig. 1**). Femoral periosteum from Pdgfrα^mT/mG^ animals (male, 2 mo old) was analyzed by immunohistochemistry at 14 and 180 d after TM administration. Nestin immunoreactivity appears red, while PDGFRα reporter activity appears green. Nuclei, DAPI (4′,6-diamidino-2-phenylindole, blue). **(c)** Percentage of dual positive PDGFRα reporter^+^Nestin^+^ cells / total PDGFRα reporter^+^ cells within periosteum at 14 and 180 d after TM labeling. **(d)** Schematic of forelimb fracture experiments, in which Pdgfrα^mT/mG^ animals underwent forelimb fracture 14 d after TM administration and were analyzed up to 14 d later (samples also shown in **Fig. 2**). **(e)** Nestin expression among PDGFRα reporter cells within the fracture callus assessed by immunohistochemistry at 3, 7, and 14 d after injury. **(f)** Percentage of dual positive PDGFRα reporter^+^Nestin^+^ cells / total PDGFRα reporter^+^ cells within the fracture callus at 3, 7, and 14 d after injury. **(g)** Schematic of mouse periosteal cell implantation, in which PDGFRα reporter^+^ (mGFP^+^) or PDGFRα reporter^-^ (Tdtomato^+^) periosteal cells are implanted subcutaneously and evaluated 56 d later (samples also shown in **Fig. 5**). **(h,i)** Nestin expression among PDGFRα reporter^+^ (GFP^+^) or PDGFRα reporter^-^ (Tdtomato^+^) periosteal implants, assessed by immunohistochemistry. Representative images **(h)** and quantification **(i)** of the frequency of Tdtomato^+^Nestin^+^ or GFP^+^Nestin^+^ cells. White dashed lines demarcate the edges of scaffold. Scale bar: 20 μm. B: bone; PO: periosteum. Dot plots represent an individual sample or animal, while whisker plots indicate mean values and one standard deviation. *In vitro* experiments were performed in biologic and experimental triplicate. N=3 animals per group. **P*<0.05; ***P*<0.01; ****P*<0.001 as assessed using two-tailed Student’s *t*-tests or one-way ANOVA with Tukey’s multiple comparisons test (a,f).


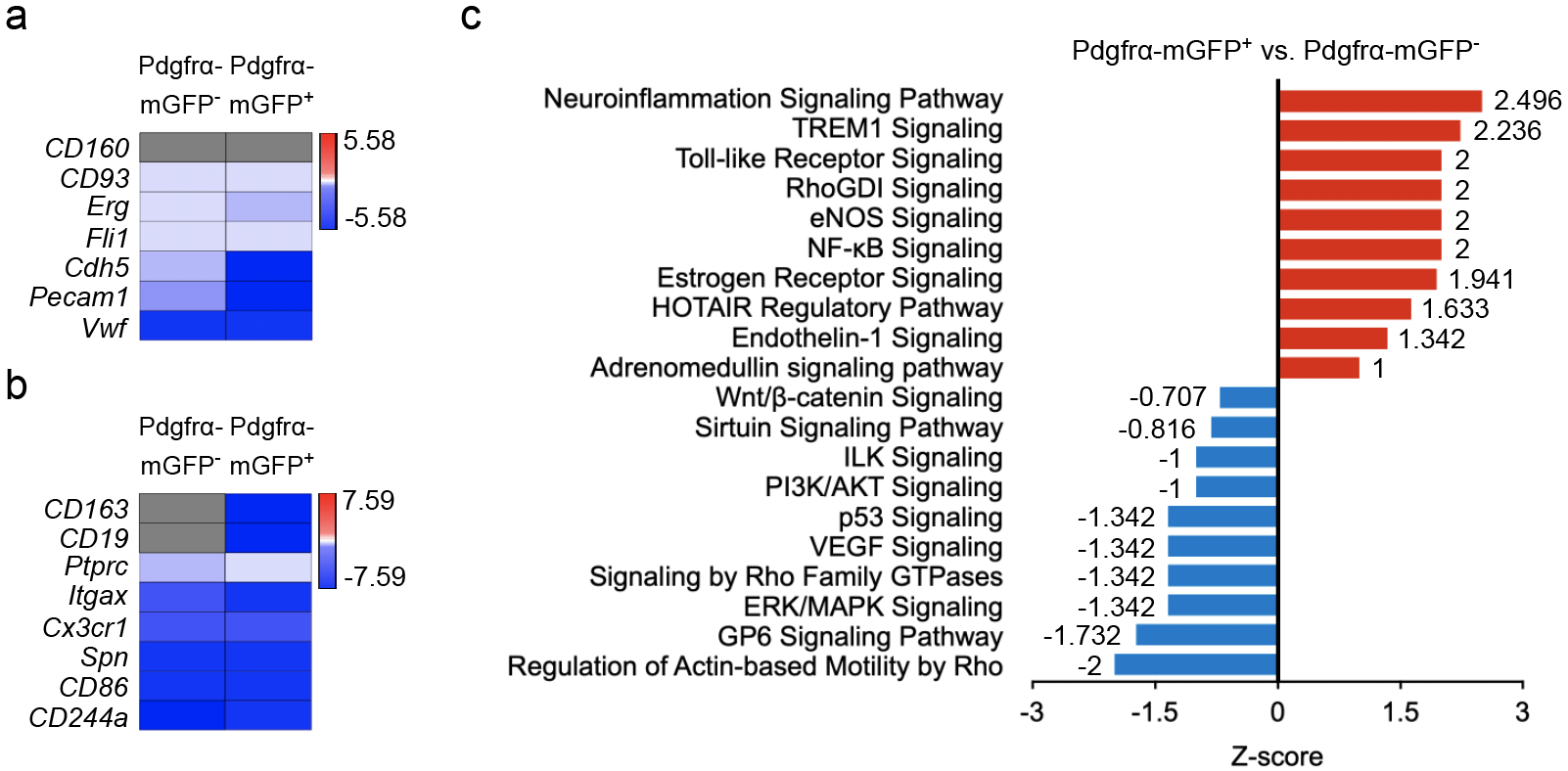


**Supplementary Figure S14. Supplemental RNA sequencing analysis of PDGFRα reporter^-/+^ periosteal cells.** Supplemental analysis for the data shown in **Fig. 7** of total RNA Sequencing of FACS purified PDGFRα reporter^-^(Tdtomato^+^)CD31^-^CD45^-^Ter119^-^ and PDGFRα reporter^+^(GFP^+^)CD31^-^CD45^-^Ter119^-^ periosteal cells from microdissected hindlimbs of Pdgfrα^mT/mG^ animals. **(a,b)** Confirmation of cell purity among FACS identified PDGFRα reporter^-/+^ periosteal cells. **(a)** Heat map demonstrating mRNA expression levels of endothelial markers among PDGFRα reporter^-/+^ periosteal cells. **(b)** Heat map of inflammatory cell markers among PDGFRα reporter^-/+^ periosteal cells. The gray color indicates no signal detected. **(c)** QIAGEN Ingenuity Pathway Analysis (IPA) identified representative pathways that were upregulated (Z-score > 0; red color) or downregulated (Z-score < 0; blue color) in PDGFRα reporter^+^ periosteal cells compared to PDGFRα reporter^-^ periosteal cells.


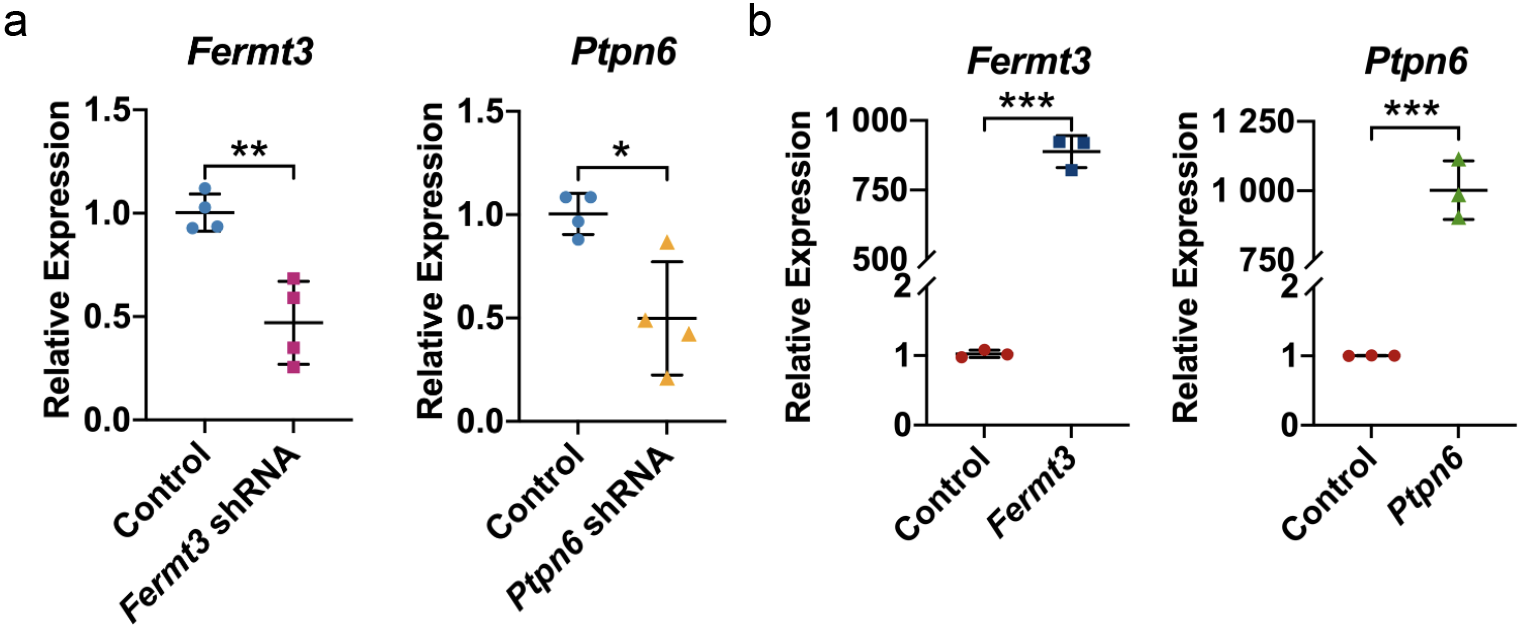


**Supplementary Figure S15. Efficiency of knockdown and overexpression of *Fermt3* and *Ptpn6* among mouse periosteal cells.** Gene expression of either *Fermt3* or *Ptpn6* as assessed by qRT-PCR after **(a)** shRNA mediated knockdown (pLKO.1-*Fermt3* shRNA or pLKO.1-*Ptpn6* shRNA) or **(b)** overexpression (pCMV6-AC-*Fermt3* or pCMV6-AC-*Ptpn6*). Dot plots represent an individual sample, while whisker plots indicate mean values and one standard deviation. **P*<0.05; ***P*<0.01; ****P*<0.001 as assessed using two-tailed Student’s *t*-tests.


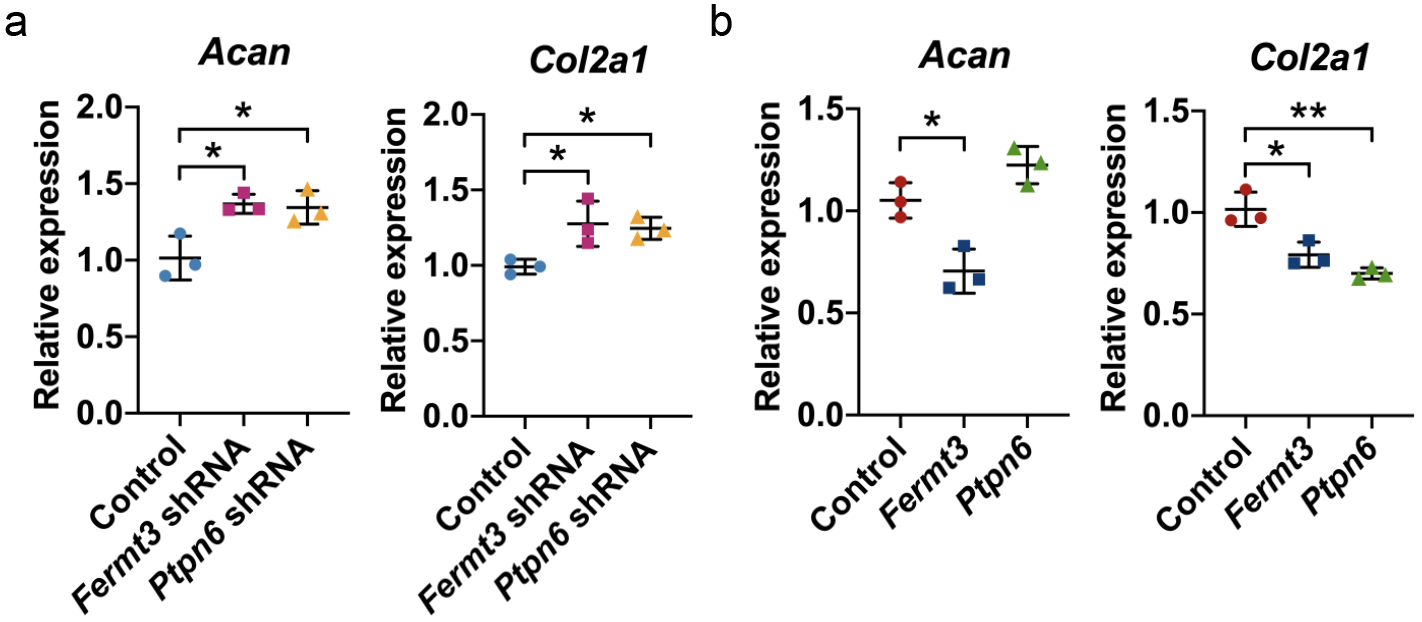


**Supplementary Figure S16. The effects of Fermt3 and Ptpn6 on chondrogenic differentiation. (a)** Chondrogenic differentiation with or without *Fermt3* or *Ptpn6* shRNA among mouse PDGFRα reporter^+^ periosteal cells assessed by chondrogenic gene expression by qRT-PCR (7 d), including *Aggrecan* (*Acan*) and *Collagen type 2 A1* (*Col2a1*). **(b)** Chondrogenic differentiation with or without *Fermt3* or *Ptpn6* overexpression in mouse PDGFRα reporter^-^ periosteal cells assessed by chondrogenic gene expression by qRT-PCR (7 d). Dot plots represent an individual sample, while whisker plots indicate mean values and one standard deviation. **P*<0.05; ***P*<0.01; ****P*<0.001 as assessed using one-way ANOVA with Tukey’s multiple comparisons test.

**Supplementary Table S1.** Frequency of PDGFRα reporter^-/+^ cells among CD31^-^CD45^-^Ter119^-^ cells from mouse periosteum by FACS sorting. Each sample represents 5-8 pooled animals.

| **Sample no.** | **PDGFRα reporter^-^ cells** | **PDGFRα reporter^+^ cells** |
| --- | --- | --- |
| 1 | 45.89 | 53.23 |
| 2 | 45.61 | 53.11 |
| 3 | 38.40 | 39.70 |
| 4 | 41.29 | 45.70 |
| 5 | 40.13 | 58.72 |
| 6 | 35.57 | 54.38 |
| Average | 41.15 | 50.81 |
| SD | 4.05 | 6.87 |

**Supplementary Table S2.** Bottom 30 differentially expressed genes among PDGFRα reporter^+^ versus PDGFRα reporter^-^ periosteal cells.

| ***Gene symbol*** | ***PDGFRα reporter^+^ vs. PDGFRα reporter^-^ Ratio*** | ***PDGFRα reporter^+^ vs. PDGFRα reporter^-^ Log2 (FC)*** | ***PDGFRα reporter^+^ vs. PDGFRα reporter^-^ -Log10 (p-value)*** |
| --- | --- | --- | --- |
| *Akap12* | 0.372063 | -1.42638 | 2.21756966 |
| *Col15a1* | 0.464007 | -1.10778 | 1.30574589 |
| *Dio3* | 0.481855 | -1.05333 | 1.73207771 |
| *Col28a1* | 0.492729 | -1.02113 | 1.72039693 |
| *Ano3* | 0.531424 | -0.912064 | 1.46430343 |
| *Ccl11* | 0.561097 | -0.833678 | 1.47595067 |
| *Slco2a1* | 0.567166 | -0.818156 | 1.91691683 |
| *Hmga1* | 0.56718 | -0.818122 | 1.92415023 |
| *Ephx2* | 0.569872 | -0.81129 | 3.3737654 |
| *Cavin2* | 0.587687 | -0.766879 | 1.5479819 |
| *Mfap5* | 0.60798 | -0.717905 | 2.09144582 |
| *Hspb7* | 0.624406 | -0.679444 | 1.72239308 |
| *Irx3* | 0.657425 | -0.605102 | 1.53622194 |
| *Itga6* | 0.661927 | -0.595256 | 1.44698886 |
| *Itgb3* | 0.670304 | -0.577113 | 1.38975091 |
| *Hspb1* | 0.686391 | -0.542897 | 1.6621715 |
| *Cryab* | 0.697352 | -0.520041 | 1.93987656 |
| *Cd80* | 0.697919 | -0.518869 | 1.42515534 |
| *Anxa3* | 0.698604 | -0.517454 | 1.67239793 |
| *Epas1* | 0.702278 | -0.509886 | 2.02228371 |
| *Adam33* | 0.705453 | -0.503378 | 2.29577825 |
| *Tbxa2r* | 0.711192 | -0.49169 | 1.33393246 |
| *Nav2* | 0.720432 | -0.473065 | 1.62360008 |
| *Rassf8* | 0.744259 | -0.426124 | 2.35673194 |
| *Emp1* | 0.747281 | -0.420277 | 2.00687649 |
| *Vcl* | 0.75183 | -0.411522 | 1.90456213 |
| *Limk1* | 0.752028 | -0.411142 | 1.42177236 |
| *Rnf157* | 0.752979 | -0.409319 | 1.39519851 |
| *Ccng1* | 0.759426 | -0.397019 | 1.57856061 |
| *Mapkapk3* | 0.762466 | -0.391255 | 1.34103897 |

**Supplementary Table S3.** Top 30 differentially expressed genes among PDGFRα reporter^+^ versus PDGFRα reporter^-^ periosteal cells.

| ***Gene symbol*** | ***PDGFRα reporter^+^ vs. PDGFRα reporter^-^ Ratio*** | ***PDGFRα reporter^+^ vs. PDGFRα reporter^-^ Log2 (FC)*** | ***PDGFRα reporter^+^ vs. PDGFRα reporter^-^ -Log10 (p-value)*** |
| --- | --- | --- | --- |
| *Pip* | 5.66357 | 2.50171 | 3.19108379 |
| *Mmp3* | 4.03175 | 2.01141 | 1.47999622 |
| *Ms4a6d* | 2.93 | 1.5509 | 2.36471937 |
| *Fermt3* | 2.53051 | 1.33943 | 1.99347834 |
| *Clec4n* | 2.47839 | 1.3094 | 1.44850121 |
| *Mmp13* | 2.28151 | 1.18999 | 2.71124119 |
| *Vegfc* | 2.12201 | 1.08543 | 2.18174441 |
| *Fap* | 2.0886 | 1.06254 | 1.31573828 |
| *Nckap1l* | 2.02757 | 1.01975 | 1.70510851 |
| *Wnt16* | 2.01404 | 1.01009 | 1.37096369 |
| *Fcgr2b* | 1.99983 | 0.999878 | 2.20436395 |
| *Pitx1* | 1.97249 | 0.980021 | 1.83602211 |
| *Mme* | 1.93 | 0.948602 | 1.34440637 |
| *Rnf128* | 1.90575 | 0.930362 | 1.34246707 |
| *Rnd1* | 1.86058 | 0.895756 | 1.32314642 |
| *Steap4* | 1.8234 | 0.866627 | 1.97480067 |
| *Ednrb* | 1.80718 | 0.853742 | 1.39941041 |
| *Cfp* | 1.68939 | 0.756499 | 1.68563763 |
| *Ptpn6* | 1.64863 | 0.721271 | 1.646078 |
| *Fxyd6* | 1.62303 | 0.698687 | 1.31539159 |
| *Alox5ap* | 1.62225 | 0.697992 | 1.71073444 |
| *Prg4* | 1.60869 | 0.685884 | 1.31378304 |
| *Cbr2* | 1.60397 | 0.68165 | 1.37370173 |
| *Prr16* | 1.53665 | 0.619788 | 1.95581315 |
| *Sfrp2* | 1.5183 | 0.602454 | 1.31793619 |
| *Mgst2* | 1.48648 | 0.571898 | 2.3386654 |
| *Ahrr* | 1.4553 | 0.541313 | 1.33007349 |
| *Tmem42* | 1.43248 | 0.518514 | 2.58993002 |
| *Gstk1* | 1.39494 | 0.480208 | 1.65720635 |
| *Lurap1* | 1.38991 | 0.474988 | 2.89042483 |

**Supplementary Table S4.** Antibodies used.

| **Antibody** | **Company** | **Catalog #** | **Use** |
| --- | --- | --- | --- |
| Mouse anti-Mouse Aggrecan | Abcam | ab3778 | IF |
| Rabbit anti-Mouse Aggrecan | NOVUS Biologicals | NB100-74350 | F |
| Mouse anti-Mouse CD31 | Abcam | ab24590 | IF |
| Mouse anti-Human CD31 | BD Pharmingen | 563653 | FACS |
| Rat anti-Mouse CD31 | BD Pharmingen | 562939 | F |
| Rat anti-Mouse CD31 | BD Pharmingen | 551262 | F/FACS |
| Mouse anti-Human CD45 | BD Pharmingen | 557833 | FACS |
| Rat anti-Mouse CD45 | BD Pharmingen | 559864 | F/FACS |
| Rat anti-Mouse CD45 | BioLegend | 103116 | F |
| Rat anti-Mouse Ter119 | BD Pharmingen | 557909 | F/FACS |
| Mouse anti-Human CD140a | BD Pharmingen | 556002 | FACS |
| Rabbit anti-Human CD140a | Abcam | ab15501 | IF |
| Goat anti-Mouse CD140a | R&D Systems | AF1062 | IF |
| Rat anti-Mouse CD140a | BD Pharmingen | 562774 | F |
| Rabbit anti-Mouse Col2 | Abcam | ab34712 | IF |
| Rabbit anti-Mouse Gli1 | Abcam | ab49314 | IF |
| Mouse Leptin R Biotinylated antibody | R&D Systems | BAF497 | IF |
| Chicken anti-Mouse Nestin | Aves Labs | NES | IF |
| Mouse anti-Human Nuclei | Sigma | MAB1281 | IF |
| Mouse anti-Mouse Osteopontin | Santa Cruz Biotechnology | sc-73631 | F |
| Rabbit anti-Human/Mouse Osteocalcin | Abcam | ab93876 | IF |
| Mouse anti-Biotin AF647 | Jackson ImmunoResearch Laboratories | 200-602-211 | IF |
| Donkey anti-Chicken Cyanine Cy™5 | Jackson ImmunoResearch Laboratories | 703-175-155 | IF |
| Donkey anti-Goat AF647 | Abcam | ab150135 | IF |
| Goat anti-Mouse AF488 | Abcam | ab150117 | IF |
| Goat anti-Mouse AF647 | Abcam | ab150119 | IF |
| Goat anti-Rabbit AF647 | Abcam | ab150079 | IF |
| Goat anti-Rabbit BV421 | BD Pharmingen | 565014 | F |
| Goat anti-Rabbit DyLight 594 | Vector Laboratories | DI-1594 | IF |
| F: Flow cytometry; FACS: Fluorescent activated cell sorting; IF: Immunofluorescent staining. | | | |

**Supplementary Table S5.** Primers used.

| Genes (Mouse) | Forward | Reverse |
| --- | --- | --- |
| *Acan* | 5’-CCTGCTACTTCATCGACCCC-3’ | 5’-AGATGCTGTTGACTCGAACCT-3’ |
| *Alp* | 5’-AGGGCAATGAGGTCACATCC-3’ | 5’-GCATCTCGTTATCCGAGTACCAG-3’ |
| *Col1a1* | 5’-TGTGTGCGATGACGTGCAAT-3’ | 5’-GGGTCCCTCGACTCCTACA-3’ |
| *Col2a1* | 5’-ACCTTGGACGCCATGAAA-3’ | 5’-GTGGACAGTAGACGGAGGAA-3’ |
| *Comp* | 5’-GGTGGTGCTCAATCAGGGAAT-3’ | 5’-CATCATCAGTGGCGGTGTTTA-3’ |
| *Fermt3* | 5’-ATGGCGGGTATGAAGACAGC-3’ | 5’-CACCAATGTGCGACTCCCC-3’ |
| *GAPDH* | 5’-GACTTCAACAGCAACTCCCAC-3’ | 5’-TCCACCACCCTGTTGCTGTA-3’ |
| *Lepr* | 5’-TGGTCCCAGCAGCTATGGT-3’ | 5’-ACCCAGAGAAGTTAGCACTGT-3’ |
| *Nestin* | 5’-CCCTGAAGTCGAGGAGCTG-3’ | 5’-CTGCTGCACCTCTAAGCGA-3’ |
| *Bglap* | 5’-AAGCAGGAGGGCAATAAGGT-3’ | 5’-TTTGTAGGCGGTCTTCAAGC-3’ |
| *Ptpn6* | 5’-GGACTTCTATGACCTGTACGGA-3’ | 5’-GCTGCGTGTAATACTCGACCA-3’ |
| *Runx2* | 5’-TGTTCTCTGATCGCCTCAGTG-3’ | 5’-CCTGGGATCTGTAATCTGACTCT-3’ |
| *Sox9* | 5’-GTGCTGAAGGGCTACGACTG-3’ | 5’-ACTTGTAATCGGGGTGGTCTT-3’ |
| *Sp7* | 5’-ATGGCGTCCTCTCTGCTTG-3’ | 5’-TGAAAGGTCAGCGTATGGCTT-3’ |

| Genes (Human) | Forward | Reverse |
| --- | --- | --- |
| *GAPDH* | 5’-CTGGGCTACACTGAGCACC-3’ | 5’-AAGTGGTCGTTGAGGGCAATG-3’ |
| *LEPR* | 5’-ACCTCTGGTTCCCCAAAAAGG-3’ | 5’-TTGGCACAGGCACAAGACAT-3’ |
| *NES* | 5’-CTGCTACCCTTGAGACACCTG-3’ | 5’-GGGCTCTGATCTCTGCATCTAC-3’ |

**Supplementary Table S6.** Animal allocation for mouse periosteal cell implantation.

| **Cell group** | **Scaffold** | **Recipient** | **Cell #** | **Implant #** |
| --- | --- | --- | --- | --- |
| Control (HA/TCP alone) | HA (18 mg) +  β-TCP (27 mg) | NOD-SCID mice, male, 8 wks | No cells | 4 |
| Mouse PDGFRα reporter^-^CD31^-^CD45^-^Ter119^-^ periosteal cells |  |  | 3.0 × 10^6^ | 6 |
| Mouse PDGFRα reporter^+^CD31^-^CD45^-^Ter119^-^ periosteal cells |  |  | 3.0 × 10^6^ | 6 |

**Supplementary Table S7.** Animal allocation for human periosteal cell implantation.

| **Cell group** | **Scaffold** | **Recipient** | **Cell #** | **Implant #** |
| --- | --- | --- | --- | --- |
| Human CD31^-^CD45^-^ PDGFRα^-^ periosteal cells | DBX Putty  (50 mg) | NOD-SCID mice, male, 8 wks | 3.0 × 10^6^ | 4 |
| Human CD31^-^CD45^-^ PDGFRα^+^ periosteal cells |  |  | 3.0 × 10^6^ | 4 |
